# Biosynthesis of UDP-α-*N*-Acetyl-d*-*mannosaminuronic Acid and CMP-β-*N*-Acetyl-d-neuraminic Acid for the Capsular Polysaccharides of *Campylobacter jejuni*

**DOI:** 10.1021/acs.biochem.3c00664

**Published:** 2024-02-21

**Authors:** Manas K. Ghosh, Frank M. Raushel

**Affiliations:** Department of Chemistry, Texas A&M University, College Station, Texas 77845, United States

## Abstract

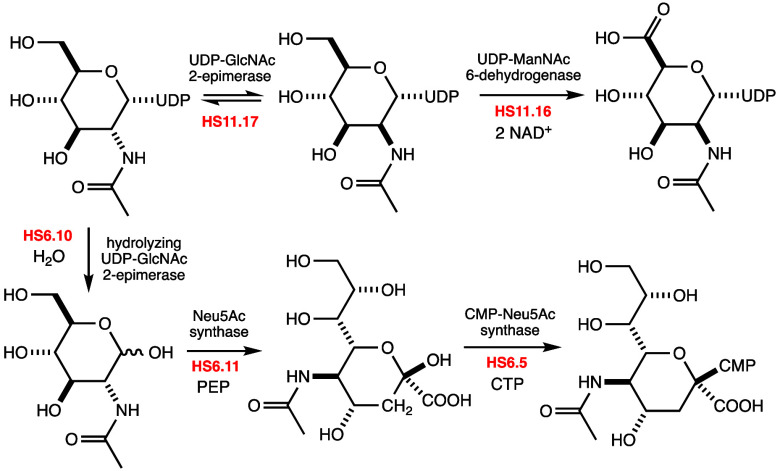

*Campylobacter jejuni* is a human pathogen and a leading cause of food poisoning in North America and Europe. The exterior surface of the bacterial cell wall is attached to a polymeric coat of sugar molecules known as the capsular polysaccharide (CPS) that helps protect the organism from the host immune response. The CPS is composed of a repeating sequence of common and unusual sugar residues. In the HS:11 serotype of *C. jejuni*, we identified two enzymes in the gene cluster for CPS formation that are utilized for the biosynthesis of UDP-α-*N*-acetyl-d-mannosaminuronic acid (UDP-ManNAcA). In the first step, UDP-α-*N*-acetyl-d-glucosamine (UDP-GlcNAc) is epimerized at C2 to form UDP-α-*N*-acetyl-d-mannosamine (UDP-ManNAc). This product is then oxidized by a NAD^+^-dependent C6-dehydrogenase to form UDP-ManNAcA. In the HS:6 serotype (*C. jejuni* strain 81116), we identified three enzymes that are required for the biosynthesis of CMP-β-*N*-acetyl-d-neuraminic acid (CMP-Neu5Ac). In the first step, UDP-GlcNAc is epimerized at C2 and subsequently hydrolyzed to form *N*-acetyl-d-mannosamine (ManNAc) with the release of UDP. This product is then condensed with PEP by *N*-acetyl-d-neuraminate synthase to form *N*-acetyl-d-neuraminic acid (Neu5Ac). In the final step, CMP-*N*-acetyl-d-neuraminic acid synthase utilizes CTP to convert this product into CMP-Neu5Ac. A bioinformatic analysis of these five enzymes from *C. jejuni* serotypes HS:11 and HS:6 identified other bacterial species that can produce UDP-ManNAcA or CMP-Neu5Ac for CPS formation.

*Campylobacter jejuni* is a Gram-negative pathogenic bacterium commonly found in the intestinal tracks of chickens and other farm animals.^1,2^*C*. *jejuni* infections in humans lead to campylobacteriosis, which can result in significant gastrointestinal consequences and in some cases in the acquisition of Guillain-Barre syndrome, an autoimmune disorder.^3−5^ The exterior surface of the *C. jejuni* cell wall is coated with a carbohydrate polymer known as the capsular polysaccharide (CPS). The CPS is composed of a specific sequence of two to five monosaccharides that can be repeated multiple times. The reducing end of the CPS is attached to a short polymer of 3-deoxy-d-*manno*-octulosonic acid (KDO), which is in turn attached to a diacyl glycerol phosphate membrane anchor.^6,7^ Different strains and serotypes of *C. jejuni* have different sequences of modified carbohydrates within the CPS. The CPS is important for bacterial cell wall stability, and deletion of the CPS biosynthesis gene cluster diminishes the pathogenicity of the organism.^8,9^

The chemical structures of the repeating monosaccharides in the CPS of at least 12 strains and serotypes of *C. jejuni* have been determined to date.^3^ In addition, the DNA sequences for the gene clusters that have been shown to contain most, but not all, of the genes for the enzymes needed to catalyze the assembly of the CPS are known for at least 33 strains and serotypes of *C. jejuni*.^10^ For example, the chemically determined structure of the repeating carbohydrate modules in the NCTC 12517 strain of *C. jejuni* (serotype HS:19) is presented in Figure 1. Here the relatively simple repeating carbohydrate module is composed of d-glucuronate (GlcA) and *N*-acetyl-d-glucosamine (GlcNAc).^11−13^ The GlcA moiety is decorated by amidation with serinol and glycosylation with l-sorbose at C2 (shown as R_2_ in Figure 1). The GlcNAc is further modified by the addition of a methyl phosphoramidate group at C4 (shown as R_1_ in Figure 1). A portion of the gene cluster for the construction of the CPS from the HS:19 serotype is presented in Figure 1.^10−13^

**Figure 1:**
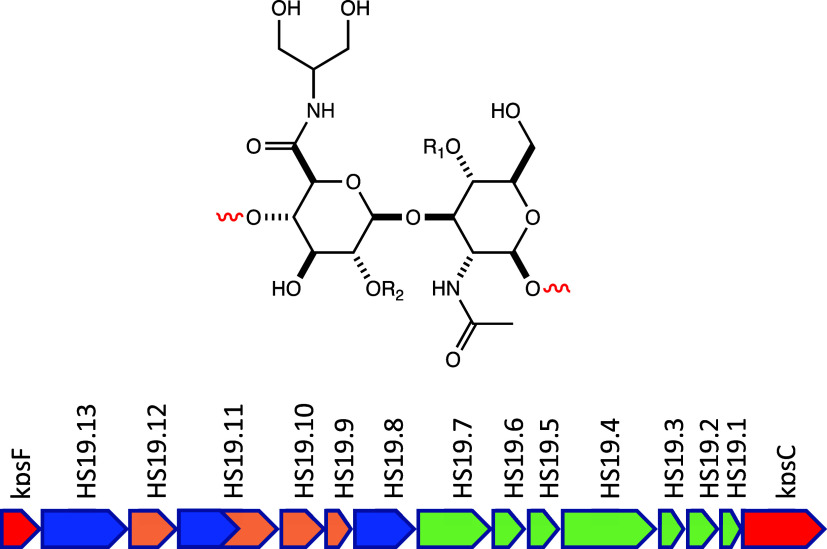
Structure of the repeating unit in the CPS from *C*. *jejuni* serotype HS:19 (top).^11−13^ The backbone of the CPS from the HS:19 serotype contains GlcA and GlcNAc. The repeating unit is further modified at C4 of the GlcNAc moiety by methyl phosphoramidation (R_1_) and at C2 of the GlcA moiety by glycosylation with l-sorbose (R_2_). Gene cluster for the enzymes required from the HS:19 serotype for the assembly of the repeating capsular polysaccharide (bottom).^10^ Additional details are provided in the text.

It has previously been demonstrated, on the basis of the corresponding enzymes functionally characterized from the HS:2 serotype, that the gene products labeled as HS19.1–HS19.7 (colored green) are responsible for the assembly and transfer of the methyl phosphoramidate modification of the GlcNAc moiety of this CPS.^14−18^ It has been further shown that the HS19.12 gene product catalyzes the NAD^+^-dependent oxidation of UDP-Glc to UDP-GlcA,^19^ HS19.10 catalyzes the PLP-dependent transamination of dihydroxy acetone phosphate to serinol phosphate,^20^ and the C-terminal half of HS19.11 catalyzes the ATP-dependent formation of the amide of glucuronic acid with serinol phosphate.^21^ The phosphate moiety of this product is ultimately hydrolyzed by the gene product of HS19.9.^22^ The catalytic properties of the putative sugar transferases (HS19.13, HS19.11, and HS19.8) have not been functionally characterized, and thus, the complete assembly process for the construction of the CPS of the HS:19 serotype has not been fully elucidated.

The repeating chemical structures for capsular polysaccharides have been determined from at least 12 different strains and serotypes of *C. jejuni*.^3^ A total of 24 different sugar moieties have thus far been identified, and these include glycerol, three pentoses (d-ribose, l-arabinose, and d-xylulose), 11 hexoses (d-glucose, d-galactose, d-mannose, d-glucitol, d-fructose, l-sorbose, d-fucose, 6-deoxy-l-altrose, d-glucuronate, *N*-acetyl-d-glucosamine, and *N*-acetyl-d-galactosamine), and nine heptoses (d-*glycero*-d-*manno*-heptose, d-*glycero*-l-*gluco*-heptose, l-*glycero*-d-*ido*-heptose, 6-deoxy-l-*galacto*-heptose, 6-deoxy-d-*ido*-heptose, 6-deoxy-d-*altro*-heptose, 6-deoxy-d-*manno*-heptose, 6-deoxy-l-*gulo*-heptose, and 3,6-dideoxy-l-*ribo*-heptose).^3,11−13,23−45^ Our efforts have been directed at improving our understanding of how individual NDP-activated monosaccharides are synthesized using the enzymes identified from the various gene clusters and the biochemical pathways for how these moieties are functionally decorated. The ultimate goal, however, is to ascertain the specific glycosyl transferases needed for the mating of the appropriate sugar donor with the proper sugar acceptor during polysaccharide formation. In this paper, we describe our efforts to interrogate the appropriate gene clusters for CPS formation from strains and serotypes of *C. jejuni* whose chemical structures have not been fully elucidated in an attempt to find additional monosaccharides that may be part of these polysaccharide chains. Here we characterize five enzymes that constitute the biochemical machinery for the formation of UDP-α*-N*-acetyl-d-mannosaminuronic acid (UDP-ManNAcA) and CMP-β-*N*-acetyl-d-neuraminic acid (CMP-Neu5Ac) during CPS assembly in two different strains/serotypes of *C. jejuni*.

## Materials and Methods

### Materials

Lysogeny broth (LB), isopropyl β-d-thiogalactopyranoside (IPTG), NAD^+^, and NADH were purchased from Research Products International. The protease inhibitor cocktail, lysozyme, DNase I, UDP-GlcNAc, ManNAc, neuraminic acid, uridine 5′-diphosphate (UDP), cytidine 5′-triphosphate (CTP), cytidine 3′,5′-cyclic monophosphate, phosphoenolpyruvate (PEP), pyruvate kinase, lactate dehydrogenase, sialic acid aldolase, pyrophosphatase, kanamycin, dithiothreitol (DTT), imidazole, and HEPES were obtained from Sigma-Aldrich. Ammonium bicarbonate, 2-mercaptoethanol, KCl, MnCl_2_, and MgCl_2_ were acquired from Sigma-Aldrich. Vivaspin 20 spin filters and HisTrap and HiTrap Q HP columns were obtained from Cytiva. The 10 kDa Nanosep spin filters were purchased from Pall Corp. (Port Washington, NY). Deuterium oxide was acquired from Cambridge Isotope Laboratories Inc., and ^18^O-labeled water (98%) was obtained from Medical Isotopes Inc.

### Equipment

Ultraviolet spectra were collected on a SpectraMax 340 (Molecular Devices) ultraviolet–visible plate reader using 96-well Greiner plates. ^1^H NMR spectra were recorded on a Bruker Avance III 400 MHz system equipped with a broad-band probe and sample changer. Mass spectrometry data were collected on a Thermo Scientific Q Exactive Focus system run in the negative ion mode.

### Plasmid Construction

The DNA for the expression of the gene for the nonhydrolyzing UDP-GlcNAc 2-epimerase (UniProt entry A0A0U3CEN8) and UDP-ManNAc 6-dehydrogenase (UniProt entry A0A0U3AB61) from *C*. *jejuni* serotype HS:11 was chemically synthesized and codon-optimized by Twist Biosciences (San Francisco, CA). Similarly, the DNA for the expression of the gene for the hydrolyzing UDP-GlcNAc 2-epimerase (UniProt entry A8FN99; C8J_1338), Neu5Ac synthase (UniProt entry A8FNA0; C8J_1339), and CMP-Neu5Ac synthase (UniProt entry A8FN94; C8J_1333) from *C*. *jejuni* serotype strain 81116 was chemically synthesized and codon-optimized by Twist Biosciences. The DNA was inserted between the NcoI and XhoI restriction sites of a pET-28a (+) expression vector. These plasmids also encode the expression of an N-terminal His_6_ affinity tag, and the complete amino acid sequences of the five proteins purified for this investigation are presented in Figures S1 and S2.

### Protein Expression and Purification

The nonhydrolyzing UDP-GlcNAc 2-epimerase and the UDP-ManNAc 6-dehydrogenase from the HS:11 serotype of *C. jejuni* were purified according to a slight modification of previously reported procedures.^40−45^ Similarly, the hydrolyzing UDP-GlcNAc 2-epimerase, Neu5Ac synthase, and CMP-Neu5Ac synthase from *C*. *jejuni* strain 81116 were expressed and purified according to a modification of previously reported procedures.^40−45^*Escherichia coli* BL21(DE3) competent cells were transformed with the appropriate plasmids. Single colonies were inoculated in 50 mL of LB medium [20 g/L yeast extract, 35 g/L tryptone, and 5 g/L sodium chloride (pH 7.0)] supplemented with 50 μg/mL kanamycin and grown at 37 °C overnight while being shaken. The starter cultures were used to inoculate 1 L of LB medium and grown at 37 °C while being shaken to an OD_600_ of ∼0.8. The culture was kept on ice for 45 min. Gene expression was induced by the addition of IPTG to a final concentration of 0.5 mM. The culture was subsequently incubated for 18 h at 14 °C while being shaken at 140 rpm. The cells were harvested by centrifugation at 7000*g* for 10 min at 4 °C, frozen in liquid N_2_, and stored at −80 °C.

The enzymes were purified at 22 °C. In a typical purification, ∼5 g of frozen cell paste was resuspended in 50 mL of buffer A [50 mM HEPES (pH 7.5), 250 mM KCl, and 5.0 mM imidazole] supplemented with 0.1 mg/mL lysozyme, 0.05 mg/mL protease inhibitor cocktail powder, 40 units/mL DNase I, and 10 mM MgCl_2_. The suspended cells were lysed by sonication, and the supernatant solution was collected after centrifugation at 10000*g* for 30 min at 4 °C. The supernatant solution was loaded onto a prepacked 5 mL HisTrap column and eluted with a linear gradient of buffer B [50 mM HEPES (pH 7.5), 250 mM KCl, and 500 mM imidazole]. Fractions containing the desired protein, as identified by sodium dodecyl sulfate–polyacrylamide gel electrophoresis (SDS–PAGE), were combined and concentrated in a 20 mL spin filter with a 30 kDa molecular weight cutoff. The imidazole was removed from the protein by dialysis using buffer C [50 mM HEPES (pH 7.5), 250 mM KCl, and 5% glycerol] at 4 °C. The protein was concentrated to 1–10 mg/mL, aliquoted, frozen in liquid N_2_, and stored at −80 °C. Typical yields of 5–10 mg for each enzyme were obtained from ∼1 L of cell culture.

### Determination of Protein Concentrations

Concentrations of the enzymes were determined spectrophotometrically using computationally derived molar absorption coefficients at 280 nm.^46^ The values of ε_280_ used for the nonhydrolyzing UDP-GlcNAc 2-epimerase and UDP-ManNAc 6-dehydrogenase from serotype HS:11 are 27 850 and 26 930 M^–1^ cm^–1^, respectively. The values of ε_280_ used for the hydrolyzing UDP-GlcNAc 2-epimerase, Neu5Ac synthase, and CMP-Neu5Ac synthase from *C. jejuni* strain 81116 were 29 800, 17 420, and 35 300 M^–1^ cm^–1^, respectively.

### Isolation of the Product Catalyzed by UDP-ManNAc C6-Dehydrogenase

The reaction catalyzed by UDP-ManNAc C6-dehydrogenase was conducted at 22 °C in 50 mM HEPES, 250 mM KCl, and 5% glycerol at pH 7.5 (or pD 7.5). The 1.0 mL reaction mixture contained 4.0 mM UDP-GlcNAc, 10 mM NAD^+^, 4.0 mM DTT, nonhydrolyzing UDP-GlcNAc C2-epimerase (6.0 μM), and UDP-ManNAc C6-dehydrogenase (6.0 μM), and the reaction performed for 18 h. The reaction was terminated by removing the two enzymes from the solution using a 0.5 mL spin filter with a 10 kDa molecular weight cutoff. The resulting flow-through was injected onto a Bio-Rad FPLC system equipped with a 5.0 mL HiTrap Q HP column. The column was washed with water, and the product eluted using a linear gradient (0% to 100%) of 500 mM NH_4_HCO_3_ (pH 8.0) over 15 column volumes. Fractions of 0.5 mL were collected and lyophilized to dryness. The resulting samples were reconstituted in either D_2_O or H_2_O and analyzed by NMR and mass spectrometry.

### Isolation of the Product from the Hydrolyzing UDP-GlcNAc C2-Epimerase

The reaction was conducted at 22 °C in 50 mM NH_4_HCO_3_ at pH 7.5 (or pD 7.5). A 1.0 mL reaction mixture containing 4.0 mM UDP-GlcNAc was incubated with the hydrolyzing UDP-GlcNAc C2-epimerase (6.0 μM) for 18 h. The reaction was terminated by removing the enzyme from the solution using a 0.5 mL spin filter with a 10 kDa molecular weight cutoff. The resulting flow-through was injected onto a Bio-Rad FPLC system equipped with a 5.0 mL HiTrap Q HP column. The flow-through was collected and lyophilized to dryness. The resulting samples were reconstituted in either D_2_O or H_2_O and analyzed by NMR spectroscopy and mass spectrometry.

### Isolation of the Product Catalyzed by the Neu5Ac Synthase

The reaction was conducted at 22 °C in 50 mM NH_4_HCO_3_ at pH 7.5 (or pD 7.5). A 1.0 mL reaction mixture containing 4.0 mM ManNAc, 8.0 mM PEP, and 10 mM MgCl_2_ was incubated with the Neu5Ac synthase (6.0 μM) for 18 h. The reaction was terminated by removing the enzyme from the solution using a 0.5 mL spin filter with a 10 kDa molecular weight cutoff. The resulting flow-through was injected onto a Bio-Rad FPLC system equipped with a 5.0 mL HiTrap Q HP column. The column was washed with water, and the product eluted using a linear gradient (0% to 100%) of 500 mM NH_4_HCO_3_ (pH 8.0) over 15 column volumes. Cytidine 3′,5′-cyclic monophosphate was used as a reference for the collection of the eluted product (net charge of −1) because the anticipated product does not absorb at a wavelength of >255 nm. Fractions of 0.5 mL were collected and lyophilized to dryness. The resulting samples were reconstituted in either D_2_O or H_2_O and analyzed by NMR spectroscopy and mass spectrometry.

### Isolation of the Product Catalyzed by CMP-Neu5Ac Synthase

The reaction was conducted at 22 °C in 50 mM HEPES and 250 mM KCl at pH 7.5. The 1.0 mL reaction mixture containing 4.0 mM Neu5Ac, 6.0 mM CTP, 10 mM MgCl_2_, and 2.5 units of pyrophosphatase was incubated with the CMP-Neu5Ac synthase (6.0 μM) for 30 min. The reaction was terminated by removing the enzyme from the solution using a 0.5 mL spin filter with a 10 kDa molecular weight cutoff. The resulting flow-through was injected onto a Bio-Rad FPLC system equipped with a 5.0 mL HiTrap Q HP column. The column was washed with water, and the product eluted using a linear gradient (0% to 100%) of 500 mM NH_4_HCO_3_ (pH 8.0) over 15 column volumes. Fractions of 0.5 mL were collected and lyophilized to dryness. The resulting samples were reconstituted in either D_2_O or H_2_O and analyzed by NMR spectroscopy and mass spectrometry.

### Determination of Kinetic Constants

The assays for the determination of the kinetic constants were conducted in a total reaction volume of 250 μL in buffer C at 25 °C. The kinetic constants for the reaction catalyzed by the nonhydrolyzing UDP-GlcNAc C2-epimerase and UDP-ManNAc C6-dehydrogenase from serotype HS:11 were determined using a coupled enzyme assay by monitoring the reduction of NAD^+^ to NADH at 340 nm.^47^ For the determination of the kinetic constants of the nonhydrolyzing C2-epimerase, the initial concentration of UDP-GlcNAc was varied between 10 μM and 5.0 mM in the presence of 2.0 mM DTT and 2.0 mM NAD^+^. The assays were conducted using 0.1 μM nonhydrolyzing C2-epimerase and a large excess of the C6-dehydrogenase coupling enzyme (10 μM). To determine the kinetic constants of the C6-dehydrogenase, the UDP-ManNAc was generated *in situ* from UDP-GlcNAc using a large excess of the nonhydrolyzing C2-epimerase (10 μM) in the presence of 2.0 mM DTT and 2.0 mM NAD^+^. The substrate concentration was varied between 10 μM and 1.5 mM. The assays were conducted with 1.0 μM C6-dehydrogenase.

The kinetic constants for the reaction catalyzed by the hydrolyzing UDP-GlcNAc C2-epimerase were determined using a coupled enzyme assay to follow the formation of UDP by monitoring the oxidation of NADH to NAD^+^ at 340 nm.^48,49^ The concentration of UDP-GlcNAc was varied between 10 μM and 1.5 mM in the presence of 0.05 μM hydrolyzing C2-epimerase, 200 units of pyruvate kinase, 250 units of lactate dehydrogenase, 2.0 mM PEP, 10 mM MgCl_2_, and 300 μM NADH. For the determination of the kinetic constants for the Neu5Ac synthase, the concentration of ManNAc was varied between 100 μM and 8.0 mM. The assays were conducted using 0.1 μM Neu5Ac synthase, 20 units of sialic acid aldolase, 250 units of lactate dehydrogenase, 2.0 mM PEP, 1.0 mM MnCl_2_, 1.0 mM DTT, and 300 μM NADH. The sialic acid aldolase catalyzes the cleavage of Neu5Ac to ManNAc and pyruvate, which is then reduced by the lactate dehydrogenase. The apparent values of *k*_cat_ and *k*_cat_/*K*_m_ were determined by fitting the initial velocity data to eq 1 using SigmaPlot 11.0
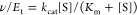
1where ν is the initial velocity of the reaction, *E*_t_ is the enzyme concentration, [S] is the substrate concentration, *k*_cat_ is the turnover number, and *K*_m_ is the Michaelis constant.

The rate of the reaction catalyzed by the CMP-Neu5Ac synthase was determined by using anion exchange chromatography to detect the formation of the CMP-Neu5Ac product. The assays were conducted with 0.05 μM CMP-Neu5Ac synthase, 1 unit of pyrophosphatase, 1.0 mM Neu5Ac, 2.0 mM CTP, and 4.0 mM MgCl_2_ in a total reaction volume of 1.0 mL. The reactions were terminated by removing the enzyme from the reaction mixture using a 0.5 mL spin filter with a 10 kDa molecular weight cutoff with time intervals of 10, 30, 60, and 120 min. The resulting flow-through was injected onto a Bio-Rad FPLC system equipped with a 5.0 mL HiTrap Q HP column. The formation of CMP-Neu5Ac was monitored at 280 nm.

### Sequence Similarity Network Analysis

Each of the FASTA protein sequences for the five enzymes from *C. jejuni* was used as the initial BLAST (Basic Local Alignment Search Tool) query in the EFI-EST webtool [Enzyme Function Initiative-Enzyme Similarity Tool (https://efi.igb.illinois.edu/efi-est/)].^50^ The sequence similarity networks (SSNs) were generated by submitting the FASTA sequences to the EFI-EST webtool. All network layouts were created and visualized using Cytoscape 3.9.1.^51^ A genome neighborhood network (GNN) was also generated using the EFI-GNT webtool (Enzyme Function Initiative-Genome Neighborhood Tool)^52^ with the 5000 protein sequences for the five enzymes as input. Using the Pfam identifiers for the five enzymes, a list of putative UDP-ManNAcA- and CMP-Neu5Ac-forming organisms was identified.

## Results and Discussion

**Figure 2:**
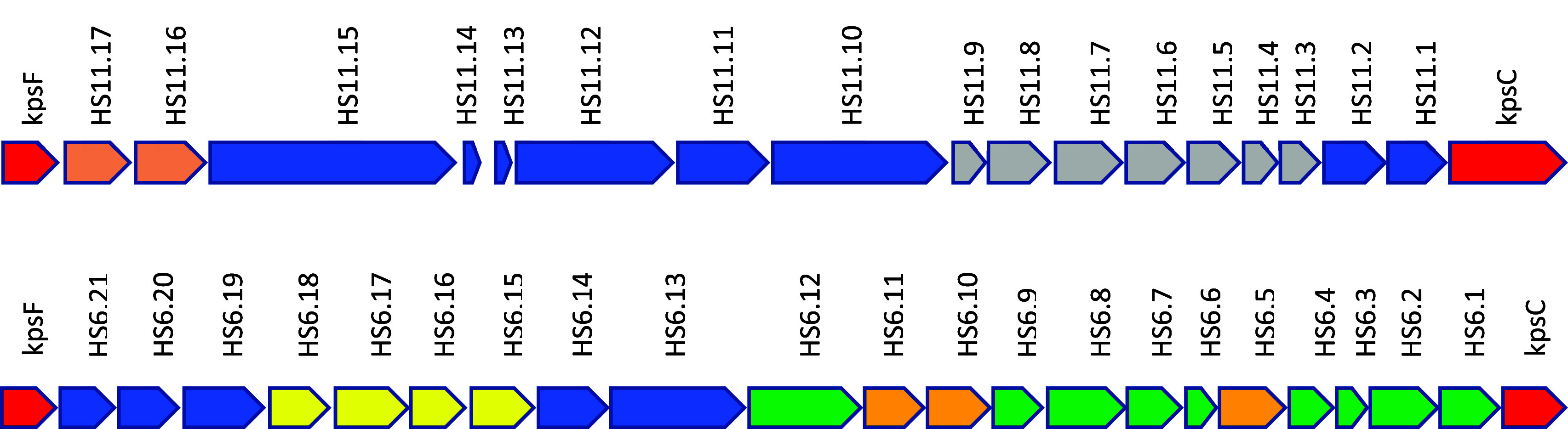
Gene clusters for the biosynthesis of the capsular polysaccharides in *C. jejuni* serotypes HS:11 (top) and HS:6 (bottom). In the gene cluster from the HS:11 serotype, it has previously been shown that the genes labeled as HS11.3–HS11.9 (colored gray) are responsible for the synthesis of GDP-3,6-dideoxy-l-*xylo*-heptose.^38,44^ Those genes colored blue represent genes for glycosyl transferases or uncharacterized enzymes. HS11.17 is a putative UDP-GluNAc 2-epimerase, while HS11.16 is a putative UDP-ManNAc 6-dehydrogenase. In the gene cluster for the HS:6 serotype, those genes colored yellow (HS6.14–HS6.17) have been shown to be responsible for the biosynthesis of UDP-l-arabinofuranoside.^56^ Those genes colored blue are for glycosyl transferases, while those colored green are of unknown function. HS6.10 likely encodes a hydrolyzing UDP-GlcNAc 2-epimerase, HS6.11 a putative Neu5Ac synthase, and HS6.5 a CMP-Neu5Ac synthase.

**Figure 3:**
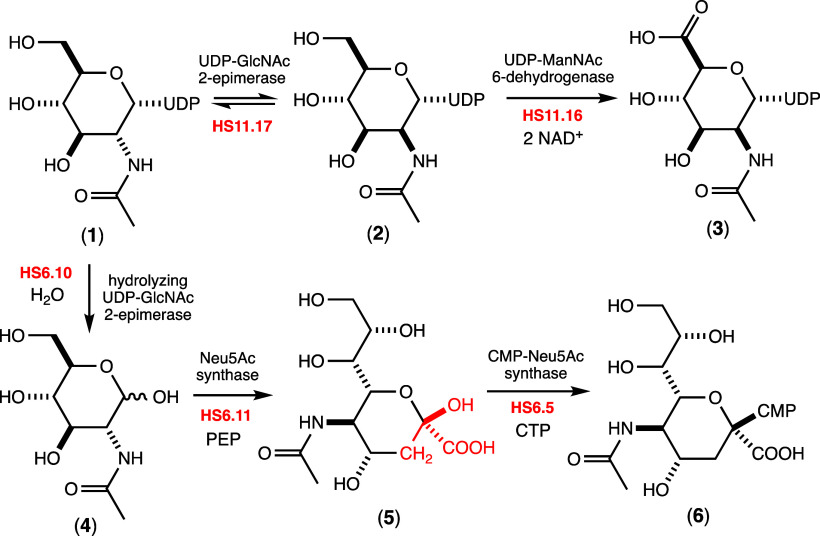
Proposed reactions catalyzed by selected enzymes for CPS formation in serotypes HS:6 and HS:11 of *C. jejuni*. The portion of Neu5Ac (compound **5**) derived from PEP in the reaction catalyzed by HS6.11 is colored red.

Our initial objective for this investigation was to identify additional sugars that will likely form part of the repeating polysaccharides in strains and/or serotypes of *C. jejuni* whose CPS structures have not been chemically characterized to date. In the proposed gene cluster for CPS formation in the HS:11 serotype, positioned between the *kpsC* and *kpsF* genes (see Figure 2), we identified a gene (HS11.17; UniProt entry A0A0U3CEN8) that is currently annotated as encoding a UDP-GlcNAc 2-epimerase. The adjacent gene (HS11.16) is provisionally annotated for the expression of a UDP-Glc 6-dehydrogenase (UniProt entry A0A0U3AB61). In this same gene cluster, we had previously characterized the enzymes (HS11.03–HS11.09) required for the biosynthesis of GDP-3,6-dideoxy-β-l-*xylo*-heptose.^38,44^ It has previously been shown by Tanner and colleagues that there are two mechanistically distinct types of UDP-GlcNAc 2-epimerases.^47,48,53−55^ The first type epimerizes C2 of UDP-GlcNAc (**1**) to make an equilibrium mixture with UDP-ManNAc (**2**), while the second also epimerizes C2 of UDP-GlcNAc but subsequently hydrolyzes the product to form ManNAc (**4**) and UDP.^47,48,53−55^ The co-localization of the 2-epimerase and the 6-dehydrogenase suggests that the first enzyme will catalyze the formation of UDP-ManNAc (**2**) from UDP-GlcNAc (**1**) while the second enzyme will catalyze the oxidation of C6 to form UDP-ManNAcA (**3**) as illustrated in Figure 3.

In the proposed gene cluster for CPS formation in *C. jejuni* strain 81116 (serotype HS:6), we identified a gene annotated for expression of a UDP-GlcNAc 2-epimerase (HS6.10, C8J_1338; UniProt entry A8FN99). Adjacent to this gene is another (HS6.11, C8J_1339; UniProt entry A8FNA0) that is annotated for the expression of Neu5Ac synthase (Figure 2). The co-localization of these two genes suggests that the first enzyme will catalyze the formation of ManNAc (**4**) while the second will result in the formation of Neu5Ac (**5**) after reaction with PEP^48,49^ as illustrated in Figure 3. A gene for the expression of the enzyme that catalyzes the formation of CMP-Neu5Ac (**6**) can also be found within this gene cluster (HS6.5, C8J_1333; UniProt entry A8FN94) to suggest that Neu5Ac (**5**) may also be a substituent of the CPS of this strain of *C. jejuni*. To provide experimental support for these observations, we set out to characterize the five enzymes that are likely responsible for the biosynthesis of the activated forms of ManNAcA (**3**) and Neu5Ac (**6**) in *C. jejuni*.

### Isolation and Functional Characterization of Five Enzymes

The five target enzymes from two different serotypes/strains of *C*. *jejuni* were produced in *E. coli* with an N-terminal polyhistidine purification tag and purified to homogeneity. These enzymes included the nonhydrolyzing C2-epimerase and C6-dehydrogenase from serotype HS:11, and the hydrolyzing C2-epimerase, Neu5Ac synthase, and CMP-Neu5Ac synthase from *C. jejuni* strain 81116.

### Reaction Catalyzed by the Nonhydrolyzing UDP-GlcNAc C2-Epimerase

**Figure 4:**
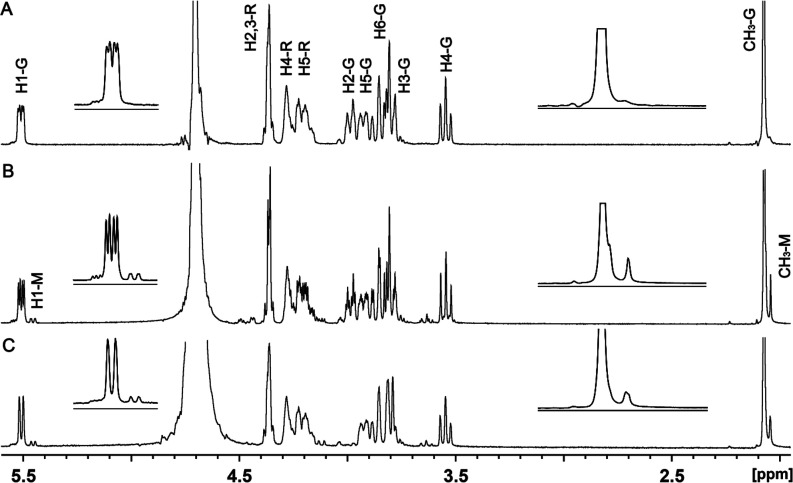
^1^H NMR spectra of UDP-ManNAc (**2**) produced with the nonhydrolyzing UDP-GlcNAc C2-epimerase from serotype HS:11. (A) UDP-GlcNAc (**1**). (B) Products of the reaction conducted in H_2_O. (C) Products of the reaction conducted in D_2_O. A portion of the ^1^H NMR spectra is shown in the insets. Resonances for the hydrogens labeled with an R correspond to the ribose moiety of UDP, while those labeled with G and M correspond to those of the GlcNAc and ManNAc moieties, respectively. In these experiments, 4.0 μM C2-epimerase was incubated with 4.0 mM compound **1** for 30 min prior to the acquisition of the NMR spectrum of the product. Additional details are provided in the text.

We first investigated the reaction catalyzed by the nonhydrolyzing UDP-GlcNAc C2-epimerase from serotype HS:11 using UDP-GlcNAc (**1**) as the initial substrate. The ^1^H NMR spectrum of this compound is shown in Figure 4a. When this substrate was incubated with the nonhydrolyzing C2-epimerase, two new resonances appeared at 2.05 and 5.46 ppm (Figure 4b). The resonance at 5.46 ppm is due to the anomeric proton (C1) of the newly formed product, UDP-ManNAc (**2**), whereas the resonance at 2.05 ppm is due to the methyl protons of this product. When the reaction was conducted in D_2_O, the resonance at 3.99 ppm for the hydrogen at C2 for the substrate and product disappears because it has been exchanged for a deuterium from the solvent due to the catalytic activity of the nonhydrolyzing C2-epimerase (Figure 4c).^47,53,54^ The C1 hydrogen (doublet of doublets) at 5.51 ppm of the substrate (**1**) is now simplified to a doublet because of the loss of ^1^H–^1^H coupling between the C1 and C2 hydrogens. An equilibrium constant for the C2-epimerase-catalyzed reaction was determined on the basis of the relative intensities of the hydrogens at C1 for the substrate (**1**) and the newly formed epimerized product (**2**). The percentages of each compound at equilibrium were calculated to be 88 and 12 for compounds **1** and **2**, respectively. The equilibrium constant from the [**2**]:[**1**] product ratio is 0.14, consistent with the previously reported value.^47,57^

**Figure 5:**
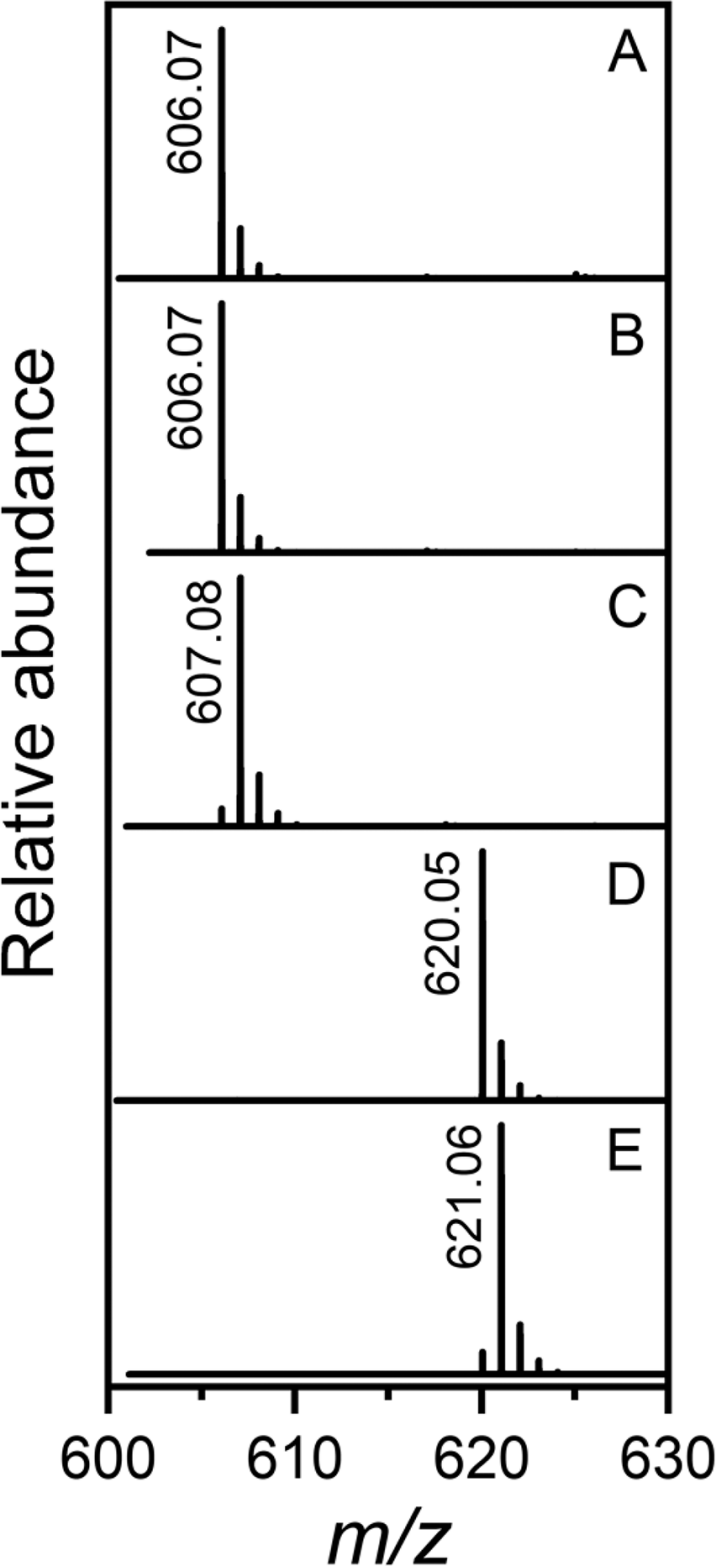
Mass spectrometric analysis of the reactions catalyzed by the nonhydrolyzing UDP-GlcNAc C2-epimerase and the UDP-ManNAc C6-dehydrogenase from serotype HS:11. (A) UDP-GlcNAc (**1**) prior to the addition of enzyme. (B) Equilibrium mixture of UDP-GlcNAc (**1**) and UDP-ManNAc (**2**) after the addition of the nonhydrolyzing C2-epimerase to compound **1** in H_2_O. (C) Same as spectrum B but with the reaction conducted in D_2_O. (D) Reaction product, UDP-ManNAcA (**3**), after the addition of the nonhydrolyzing C2-epimerase and C6-dehydrogenase to compound **1** in H_2_O. (E) Same as spectrum D but with the reaction conducted in D_2_O.

The incorporation of one deuterium in the nonhydrolyzing C2-epimerase reaction product was also confirmed using mass spectrometry. ESI-MS (negative ion mode) of UDP-GlcNAc (**1**) before the addition of the C2-epimerase is shown in Figure 5a with an ion at *m*/*z* 606.07 for the M – H anion. ESI-MS of the UDP-GlcNAc/UDP-ManNAc equilibrium mixture with an ion at *m*/*z* 607.08 for the M – H anion was obtained after incubation of **1** with the nonhydrolyzing C2-epimerase in D_2_O (Figure 5c), consistent with the addition of one deuterium atom in the substrate and product. The control reaction was conducted in H_2_O (Figure 5b).

### Reaction Catalyzed by UDP-ManNAc C6-Dehydrogenase

**Figure 6:**
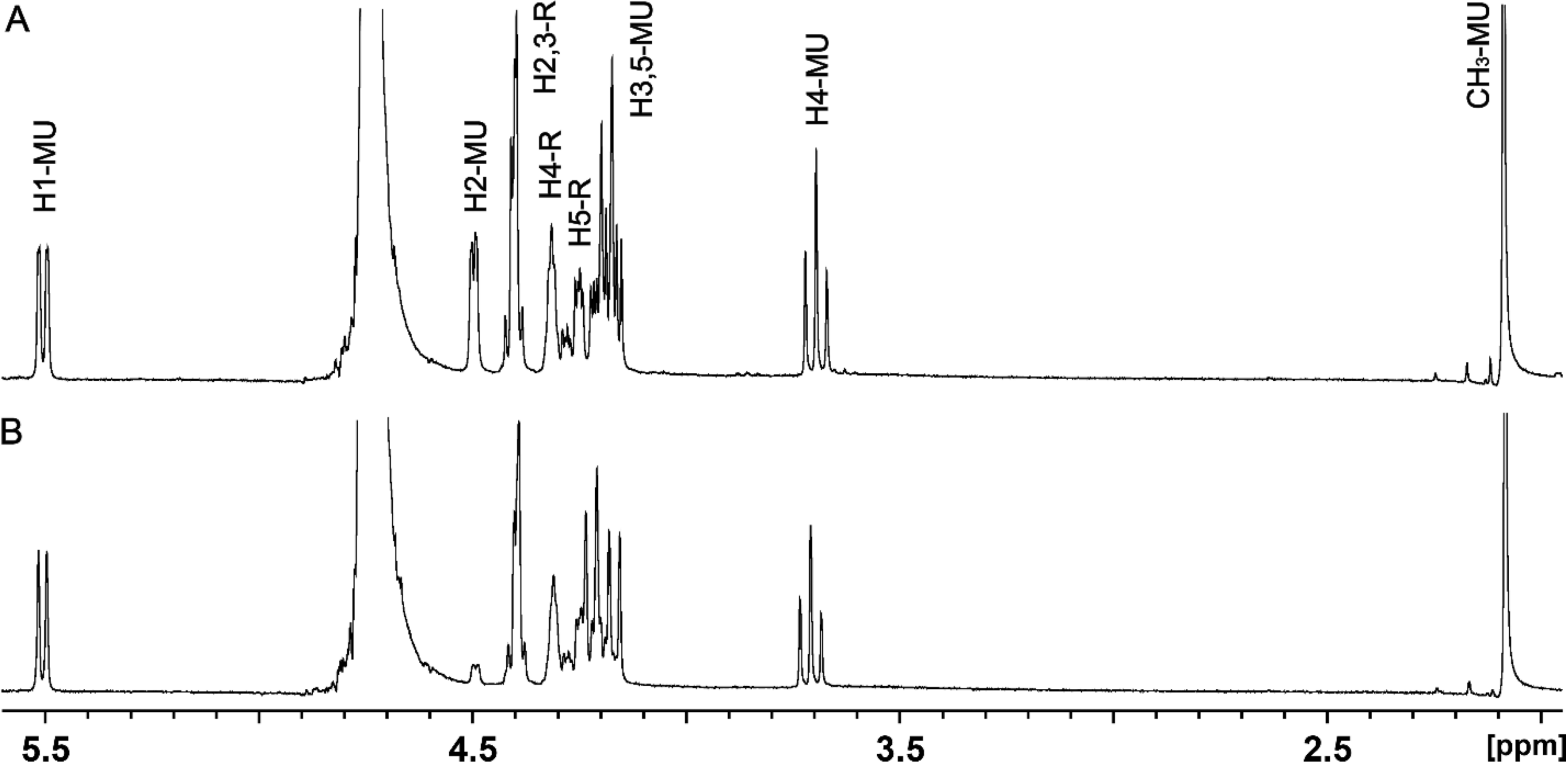
^1^H NMR spectra of UDP-ManNAcA (**3**) produced with the catalytic activities of nonhydrolyzing C2-epimerase and C6-dehydrogenase from serotype HS:11 using UDP-GlcNAc (**1**) as the initial substrate. (A) Reaction conducted in H_2_O. (B) Reaction conducted in D_2_O. The resonances for the hydrogens labeled with an R correspond to those of the ribose moiety of UDP, while those labeled with a MU correspond to those of the ManNAcA moiety. Additional details are provided in the text.

The reaction catalyzed by UDP-ManNAc C6-dehydrogenase from serotype HS:11 was initiated using UDP-GlcNAc (**1**) as the starting substrate. When **1** was incubated with the nonhydrolyzing C2-epimerase and the C6-dehydrogenase in the presence of NAD^+^, a new compound was formed, and its ^1^H NMR spectra are provided in Figure 6a and Figure S3. When the reactions were conducted in D_2_O, the resonance for the hydrogen at C2 disappears in the ^1^H NMR spectrum because it has been exchanged for the deuterium from the solvent due to the catalytic activity of the nonhydrolyzing C2-epimerase (Figure 6b and Figure S4). The assignment of resonances in the NMR spectra is based on the two-dimensional (2D) COSY NMR spectrum and the loss of signal for the hydrogen at C2 when the rection is conducted in D_2_O.

The reaction product was further confirmed using mass spectrometry. The M−H anion for UDP-NAcA (**3**) was identified at *m*/*z* 620.05 by ESI-MS after incubation of **1** with the nonhydrolyzing C2-epimerase and C6-dehydrogenase in the presence of NAD+ (Figure 5d). When the reaction was conducted in D_2_O, ESI-MS of the product exhibits an ion at *m*/*z* 621.06 for the M – H anion, consistent with the addition of one deuterium atom at C2 (Figure 5e).

### Reaction Catalyzed by the Hydrolyzing UDP-GlcNAc C2-Epimerase

**Figure 7:**
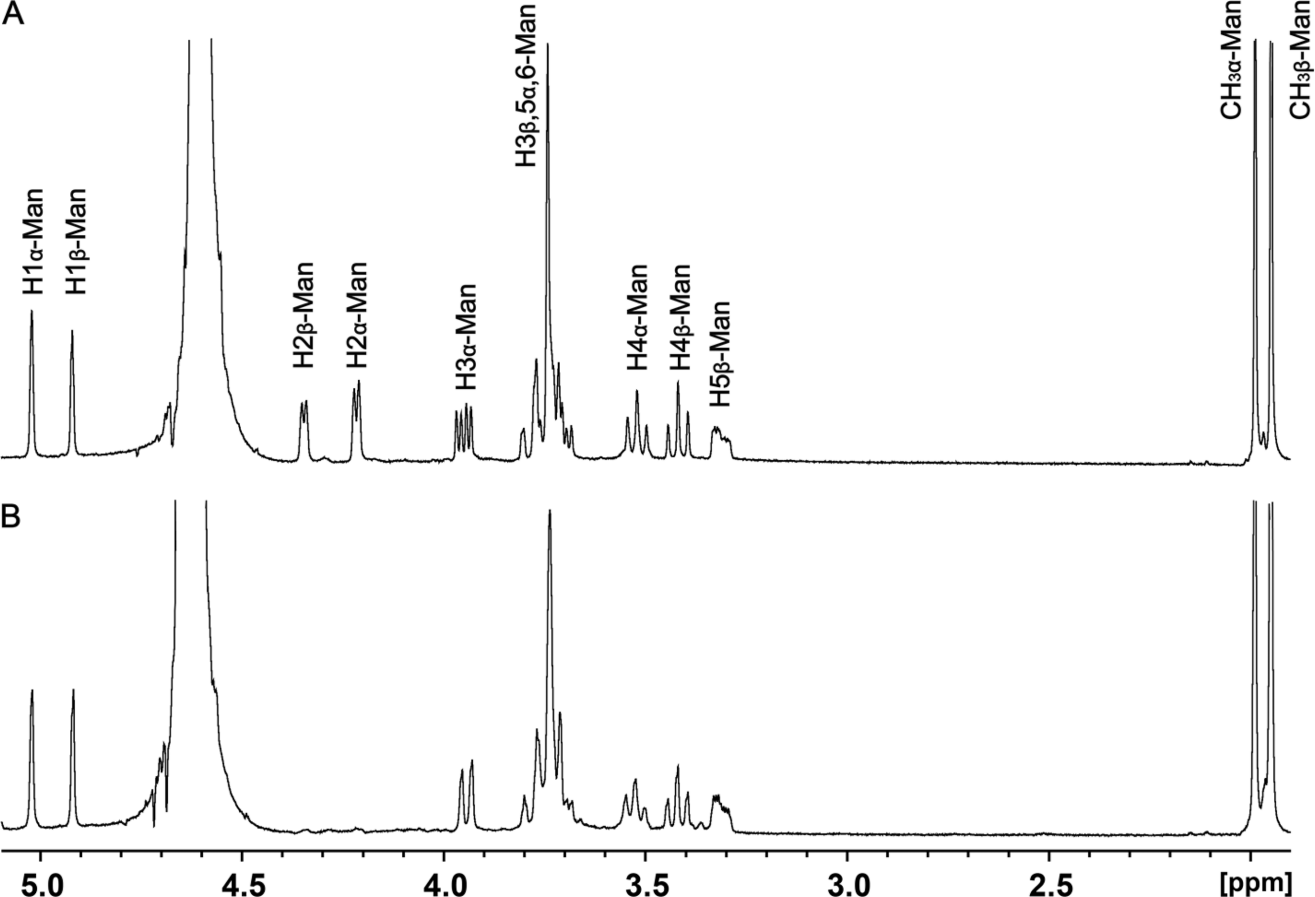
^1^H NMR spectra of ManNAc (**4**) produced with the hydrolyzing C2-epimerase from *C. jejuni* strain 81116. (A) Reaction conducted in H_2_O. (B) Reaction conducted in D_2_O. Additional details are provided in the text.

The reaction catalyzed by the putative hydrolyzing C2-epimerase from *C. jejuni* strain 81116 (HS:6) was tested using UDP-GlcNAc (**1**) as the initial substrate. When this compound was incubated with the hydrolyzing C2-epimerase, two new resonances appeared at 5.09 and 4.99 ppm (Figure 7a and Figure S5). The signals at 5.09 and 4.99 ppm correspond to the α and β anomers, respectively, of the newly formed ManNAc (**4**). When the reaction was conducted in D_2_O, the resonances for the hydrogen at C2 disappear because they have been exchanged for deuterium from the solvent due to the catalytic activity of the hydrolyzing C2-epimerase (Figure 7b and Figure S6). At equilibrium, the ratio of the anomers is 1:1, consistent with previous reports.^48,55^ The assignment of resonances in the NMR spectra was based on the 2D COSY NMR spectrum and the loss of signals for the hydrogen at C2 when the reaction was conducted in D_2_O.

The incorporation of one deuterium in the hydrolyzing C2-epimerase catalyzed product was confirmed using mass spectrometry. ESI-MS (negative ion mode) of the product prepared in H_2_O, ManNAc (**4**), is shown in Figure S7a with an ion at *m*/*z* 220.08 for the M – H anion. The M−H anion for ManNAc (**4**) with a deuterium at C2 was identified at *m*/*z* 221.08 after incubation of **1** with the hydrolyzing C2-epimerase in D_2_O (Figure S7b). When the reactions are conducted in 50% D_2_O, ESI-MS of the product (Figure S7c) confirms the formation of ManNAc (**4**) with *m*/*z* values of 220.08 and 221.08 and an intensity ratio of ∼1:1. Similarly, when the reaction steps are conducted in 50% H_2_^18^O, ESI-MS of product ManNAc (**4**) is shown in Figure S7d at *m*/*z* values of 220.08 and 222.08 with an intensity ratio of ∼1:1. Reaction mechanisms for the hydrolyzing and nonhydrolyzing 2-epimerases are shown in Scheme S1.^47,48,53−55^ The exchange of solvent deuterium at C2 for both enzymes and the incorporation of ^18^O from solvent in the hydrolyzing C2-epimerase is consistent with the formation of a 2-acetamidoglucal intermediate as proposed by Tanner and colleagues.^47,48,53−55^

### Reaction Catalyzed by *N*-Acetylneuraminate Synthase

The reaction catalyzed by the putative Neu5Ac synthase was tested using ManNAc (**4**) and PEP as the likely substrates. When ManNAc (**4**) was incubated with the synthase in the presence of PEP, a new compound was formed whose ^1^H NMR spectra are provided in Figure S8 for the equilibrium mixture of the α and β anomers. The newly formed compound, Neu5Ac (**5**), shows resonances at 2.30 and 1.87 ppm due to the methylene group at C3. The hydrogens from C3 to C9 were assigned on the basis of the 2D COSY NMR spectrum and comparison with previously published spectra.^49,58^ The reaction product was confirmed by mass spectrometry. ESI-MS (negative ion mode) of the product Neu5Ac (**5**) is shown in Figure S10a with an ion at *m*/*z* 308.09 for the M – H anion, consistent with previous reports.^49^ Identical ^1^H NMR (Figure S9) and mass (Figure S10b) spectra were obtained when the reactions were conducted in D_2_O, indicating the lack of solvent exchange during the enzyme-catalyzed reaction.

### Reaction Catalyzed by CMP-Neu5Ac Synthase

We investigated the reaction catalyzed by the putative CMP-Neu5Ac synthase using CTP as the nucleotide donor. When Neu5Ac (**5**) was incubated with CTP in the presence of MgCl_2_ and an enzyme, a new compound was identified after purification by anion exchange chromatography. The results are consistent with the formation of CMP-Neu5Ac (**6**). The identity of the new product, CMP-Neu5Ac, was confirmed by NMR spectroscopy (Figure S11) and mass spectrometry (Figure S12). The signal at 5.99 ppm in the ^1^H NMR spectrum corresponds to the hydrogen at C1 of the ribose ring of the newly formed compound, CMP-Neu5Ac (**6**). The ^1^H NMR and ^1^H–^1^H COSY spectra of CMP-Neu5Ac (**6**) are shown in Figure S11. The formation of CMP-Neu5Ac (**6**) was further supported by ESI-MS. A peak at *m*/*z* 613.14 was observed that is consistent with the expected mass for CMP-Neu5Ac (**6**).

### Kinetic Constants for the Enzyme-Catalyzed Reactions

The kinetic constants for the enzymes interrogated for this investigation, except for CMP-Neu5Ac synthase, were determined spectrophotometically at 340 nm by monitoring the change in concentration of NADH or NAD^+^. The kinetic constants are listed in Table 1.

**Table 1:** Steady State Kinetic Parameters for Nonhydrolyzing C2-Epimerase, C6-Dehydrogenase, Hydrolyzing C2-Epimerase, *N*-Acetylneuraminate Synthase, and CMP-Neu5Ac Synthase^a^

enzyme	*k*_cat_ (s^–1^)	*K*_m_ (μM)	*k*_cat_/*K*_m_ (M^–1^ s^–1^)
nonhydrolyzing UDP-GlcNAc C2-epimerase	5.7 ± 0.2	1370 ± 50	4200 ± 200
UDP-ManNAc C6-dehydrogenase	0.20 ± 0.01	22 ± 1	8700 ± 400
hydrolyzing UDP-GlcNAc C2-epimerase	3.1 ± 0.1	160 ± 10	19900 ± 1000
Neu5Ac synthase	1.7 ± 0.1	2100 ± 200	800 ± 50
CMP-Neu5Ac synthase	1.2 ± 0.2	not determined	not determined

a At pH 7.5 and 25 °C.

### Bioinformatic Analysis

The SSN of the 5000 closest homologues of the nonhydrolyzing C2-epimerse from *C*. *jejuni* serotype HS:11 is shown in Figure S13 at a sequence identity cutoff of 59%. In this SSN, there are two previously characterized enzymes (pink circles), and these include the nonhydrolyzing C2-epimerase from *E. coli* K12 and *Neisseria meningitidis* DSM15465. These enzymes have sequence identities of 57% and 52%, respectively, with the corresponding enzyme from *C*. *jejuni* serotype HS:11 (yellow circle). These two proteins have been shown to catalyze the C2 epimerization of UDP-GlcNAc to form UDP-ManNAc in both organisms.^47,59^ Similarly, the SSN of the 5000 closest homologues of UDP-ManNAc C6-dehydrogenase from *C*. *jejuni* serotype HS:11 is shown in Figure S14 at a sequence identity cutoff of 73%. In this SSN, there is one previously partially characterized enzyme (pink circle) from *E. coli* K12.^47^ This enzyme has a sequence identity of 63% with the corresponding enzyme from *C*. *jejuni* serotype HS:11 (yellow circle).

To further understand the protein pairs necessary for the formation of UDP-ManNAcA (**3**) across various organisms, a genome neighborhood network (GNN) was created using the 5000 protein sequences identified in the SSNs from Figures S13 and S14 as the initial input. The genome neighborhood was filtered by protein pairs that contained the Pfam identifier for the nonhydrolyzing C2-epimerase (PF02350) and C6-dehydrogenase (PF00984) from *C. jejuni*. A total of 1888 protein pairs for the nonhydrolyzing C2-epimerase and C6-dehydrogenase (PF00984) were identified (Figure S15). We predict that these pairs of proteins are responsible for the biosynthesis of UDP-ManNAcA from >1000 other organisms. This preliminary bioinformatic analysis demonstrates that the biosynthesis of UDP-ManNAcA^3^ has not been functionally characterized in many bacterial systems to date but that the two enzymes needed for the formation of this compound can be found in a diverse set of bacterial species.

The SSNs of the 5000 closest homologues of the hydrolyzing C2-epimerase, Neu5Ac synthase, and CMP-Neu5Ac synthase from *C*. *jejuni* serotype HS:6 are shown in Figures S16–S18 at sequence identity cutoffs of 50%, 50%, and 55%, respectively. In these SSNs, previously characterized enzymes are shown as pink circles, and the corresponding enzymes from *C*. *jejuni* serotype HS:6 are colored yellow. These enzymes together catalyze the formation of CMP-Neu5Ac.^48,49,55,60,61^

To further understand the cluster of enzymes necessary for the formation of CMP-Neu5Ac across various organisms, a genome neighborhood network was created using the 5000 protein sequences identified in the SSN from Figures S16–S18 as the initial input. The genome neighborhoods were filtered by the three enzymes that contained the Pfam identifier for the hydrolyzing C2-epimerase (PF02350), Neu5Ac synthase (PF03102), and CMP-Neu5Ac synthase (PF02348) from *C. jejuni*. More than 1000 enzyme clusters containing the hydrolyzing C2-epimerase (PF02350), Neu5Ac synthase (PF03102), and CMP-Neu5Ac synthase (PF02348) were identified (Figure S19). Like the biosynthesis of UDP-ManNAcA (**3**), the three enzymes required for the biosynthesis of CMP-Neu5Ac (**6**) from UDP-GlcNAc (**1**) have not been widely characterized, but they can be found clustered together in a wide variety of bacterial species.

## Conclusions

The biosynthetic pathways for the assembly of nucleotide-activated ManNAcA (**3**) and Neu5Ac (**6**) from two different serotypes of the human pathogen *C. jejuni* were determined. We identified two genes in the gene cluster for the biosynthesis of the CPS in the HS:11 serotype of *C. jejuni* that were utilized to convert UDP-GlcNAc (**1**) into UDP-ManNAcA (**3**). In the first step, **1** is converted into UDP-ManNAc (**2**). This product is then oxidized by a NAD^+^-dependent C6-dehydrogenase to form UDP-ManNAcA (**3**). We also identified three enzymes in the putative gene cluster for the biosynthesis of CPS in *C. jejuni* strain 81116 (HS:6) that were used to convert UDP-GlcNAc (**1**) into CMP-Neu5Ac (**6**). In the first step, **1** is converted into ManNAc (**4**). This product is then condensed with PEP by Neu5Ac synthase to form Neu5Ac (**5**). In the final step, **5** is converted into CMP-Neu5Ac (**6**). However, the CPS for the HS:6 serotype has been reported to include d-glucose, d-glucuronic acid, and d-mannose.^27^ The presence of Neu5Ac and l-arabinose (from the catalytic action of HS6.14–HS6.17) has not yet been identified.^27^ We are presently working to understand this ambiguity more fully.

## Supporting Information Available

Amino acid sequences of the purified proteins, the SSN and GNN of the proteins, and NMR and mass spectra of carbohydrate substrates and products (PDF)

## Accession Codes

A0A0U3CEN8 for nonhydrolyzing C2-epimerase from serotype HS:11, A0A0U3AB61 for C6-dehydrogenase from HS:11, A8FN99 for hydrolyzing C2-epimerase from serotype HS:6, A8FNA0 for *N*-acetylneuraminate synthase from HS:6, and A8FN94 for CMP-*N*-acetylneuraminate synthase from HS:6.
