# Supplementary material for: Biosynthesis of UDP-α-N-Acetyl-d-mannosaminuronic Acid and CMP-β-N-Acetyl-d-neuraminic Acid for the Capsular Polysaccharides of Campylobacter jejuni
Source: Biochemistry. 2024 Feb 21;63(5):688–98. doi: 10.1021/acs.biochem.3c00664 (PMC10919079; doi:10.1021/acs.biochem.3c00664)
Supplement: Supplementary file 1 — bi3c00664_si_001.pdf [file bi3c00664_si_001.pdf]

Supporting Information

Biosynthesis of UDP-α-*N*-Acetyl-D-mannosaminuronic Acid and CMP-β-*N*-acetyl-D-neuraminic Acid for the Capsular Polysaccharides of *Campylobacter jejuni*

Manas K. Ghosh[‡, §], and Frank M. Raushel[‡,*]

[‡]Department of Chemistry, Texas A&M University, College Station, Texas 77845 US

[§]**Present Address**

Manas K. Ghosh – Department of Chemistry, Sovarani Memorial College (University of Calcutta), Jagatballavpur, West Bengal, India 711408.

*To whom correspondence may be sent: raushel@tamu.edu

(A)
MGSSHHHHHHSSGENLYFQGGHMVKKILIVFGTRPEAIKMAPLVKIMENRNDVNFRVCVTGQHR
QMLDQILNIFDIKPDYDLNIMQENQDLYDITLKILCGMKNVLNDFKPNIVLVHGDTTTASVTALAA
FYQKIKIAHVEAGLRTYNICNPWPEEANRQIIGVLANIHFTPTVKSAENLIKEGKNKEGIFITGNTVI
DALFYMTEKIKNDKTFKIKVLSSIGNEYKINDNKKFILVTGHRRENFGEGFLQICEALKIIAINNPDI
DIVYPVHLNPNVQKPVKMLLLNISNIYLINPLKYEEFIYLMSKSYFIITDSGGIQEEAPSLGKPILVMR
ETTERPEAVEAGVVKLVGACKQNIIRESQKLIDDQDEYEKMSKAYNPYGDGRACEKIINILIKGKNN
ESI

(B)
MGSSHHHHHHSSGENLYFQGHMNQFNKVCVIGLGYIGLPTAAIFASRKIKVIGVDINQNIVDIINKG
KIHIVEPGLDILVHMVVNDGYLKATTLPEEADAFIIAVPTPFKGDNHEPNLDYIEMASKAIAKVLKK
GNLVILESTSPVGTTEQMAKWLAEERSDLSFPHQCGEVSDIKIAHCPERVLPGQVIRELVENDRIIG
GMTQKCAEYAIRLYKIFVQGECIMTNARTAEMTKLTENSFRDLNIAFANELSILCDKLDINVWELI
KLANRHPRVNILQPGCGVGGHCIAVDPWFIVYQNPNEAKIIKTAREVNDNKPNFVVQKIKKKIKDI
LGPKIACLGLAFKPDIDDLRESPALDIVIKLASENASQILVVEPNIKQLPLKLQNKRNIKLVCLSQAL
DEADVVAVLVRHKEFIEVQSDKVVSFC

Figure S1. The protein sequences are shown for the non-hydrolyzing UDP-GlcNAc 2-epimerase (UniProt entry: A0A0U3CEN8) (A) and UDP-ManNAc 6-dehydrogenase (UniProt entry: A0A0U3AB61) (B) from *C. jejuni* serotype HS:11. The residues added by the polyhistidine tag are highlighted in red.

(A)
MGSSHHHHHHSSGENLYFQGGHMKKILFITGTRADYSKIKSLMYRVQNSSEFELCIFATGMHLSK
NFGYTVKELYKNGFKNIYEFINYDKYYQTDKALATTIDGFSRYVNELKPDLIVVHGDRIEPLAAAIV
GALNNILVAHIEGGEISGTIDDSLRHAISKLAHIHLVNDEFAKKRLMQLGEDEKSIFIIGSPDLELLN
DNKISLNEAKKYYDINYENYALLMFHPVTTEITSIKNQADNLVKALIQSNKNYIVIYPNNDLGFELI
LQSYEELKNNPRFKLFPSLRFEYFITLLKNADFIIGNSSCILKEALYLKTAGILVGSRQNGRLGNENT
LKVNANSDEILKAINTIHKKQDLFSAKLEILDSSKLFFEYLQSGEFFKLSTQKVFKDIII

(B)
MGSSHHHHHHSSGENLYFQGHMKEIKIQNIIINEEKAPLVVPEIGINHNGSLELAKIMVDAAFSAGA
KIIKHQTHIVEDEMSKAAKKVIPGNAKISIYEIMQKCALDYKDELALKEYTEKLGLVYLSTPFSRAG
ANRLEDMGVSAFKIGSGECNNYPLIKHIAAFKKPMIVSTGMNSIESIKPTVKILLDNEIPFVLMHTT
NLYPTPHNLVRLNAMLELKKEFSCMVGLSDHTTDNLACLGAVALGACVLERHFTDSMDRSGPDI
VCSMDTKALKELIMQSEQMAIMRGNNESKKAAKQEQVTIDFAFASVVSIKDIKKGEVLSMDNIWV
KRPGLGGISAAEFENILGKKALRDIENDTQLSYEDFA

(C)
MGSSHHHHHHSSGENLYFQGHMVIAVIPARSGSKGIKNKNLVLLNNQPLIYYTIKAALNSKCISKVL
VSSDSEEILSYAKSQNVDILKRPIELAQDDTTSDKVLLHALEFYKDYEDVIFLQPTSPLRTNIHIDEA
FKIYKNSDANALISVTECDNKILKAFICDNKGDLKGICDDEYPFMPRQKLPKTYMSNGAIYILSIKDF
LNKPSFLQSKTKYFLMNKISSLDIDNLEDLKQVENIQKMKGCVVWISGLAGAGKTTISSGLYKKLK
EKYNNSVLLDGDELRKIFKHTGYTREERLESAKKISSLCSFLAKNDIIVICATISLFEEIYLLNRNTIE
NYFEVFVDCPMEELILRDQKGLYSGALKGEIKDVVGVDIKYAKPNAHYIIDNSSKTDLEKKINNLYN
EVELFFNKER

Figure S2. The protein sequences are shown for hydrolyzing UDP-GlcNAc 2-epimerase (UniProt entry: A8FN99; C8J_1338) (A), Neu5Ac synthase (UniProt entry: A8FNA0; C8J_1339) (B), and CMP-Neu5Ac synthase (UniProt entry: A8FN94; C8J_1333) (C) from *C. jejuni* strain 81116. The residues added by the polyhistidine tag are highlighted in red.

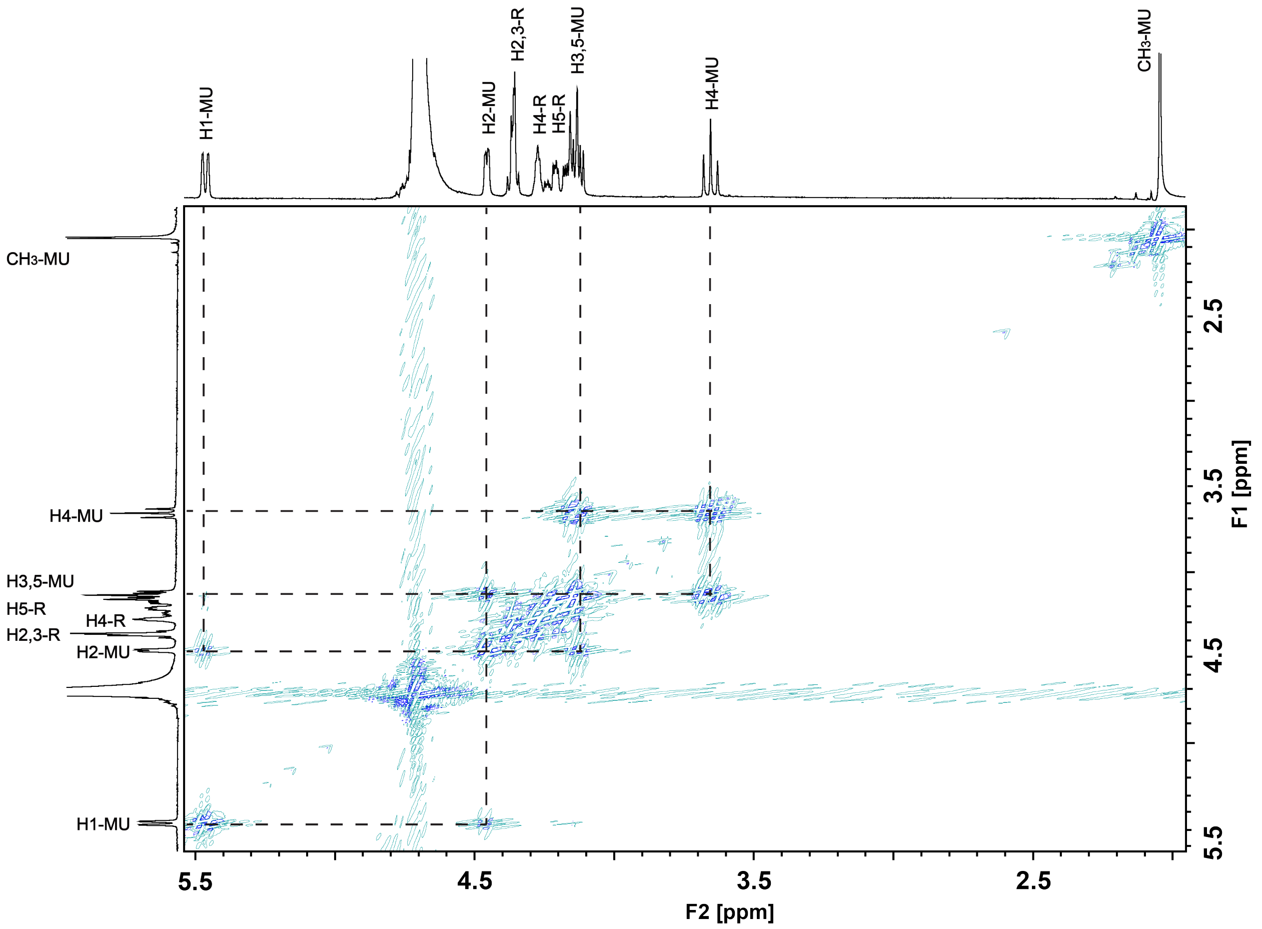

Figure S3. ^{1}H-^{1}H COSY NMR spectra of UDP-ManNAcA (**3**) produced in H_2O with the catalytic activities of non-hydrolyzing C2-epimerase and C6-dehydrogenase from serotype HS:11. Resonances for the hydrogens labeled with a "R" correspond to the ribose moiety of UDP, while those labeled with a "MU" correspond to those of the ManNAcA moiety. Additional details are provided in the text.

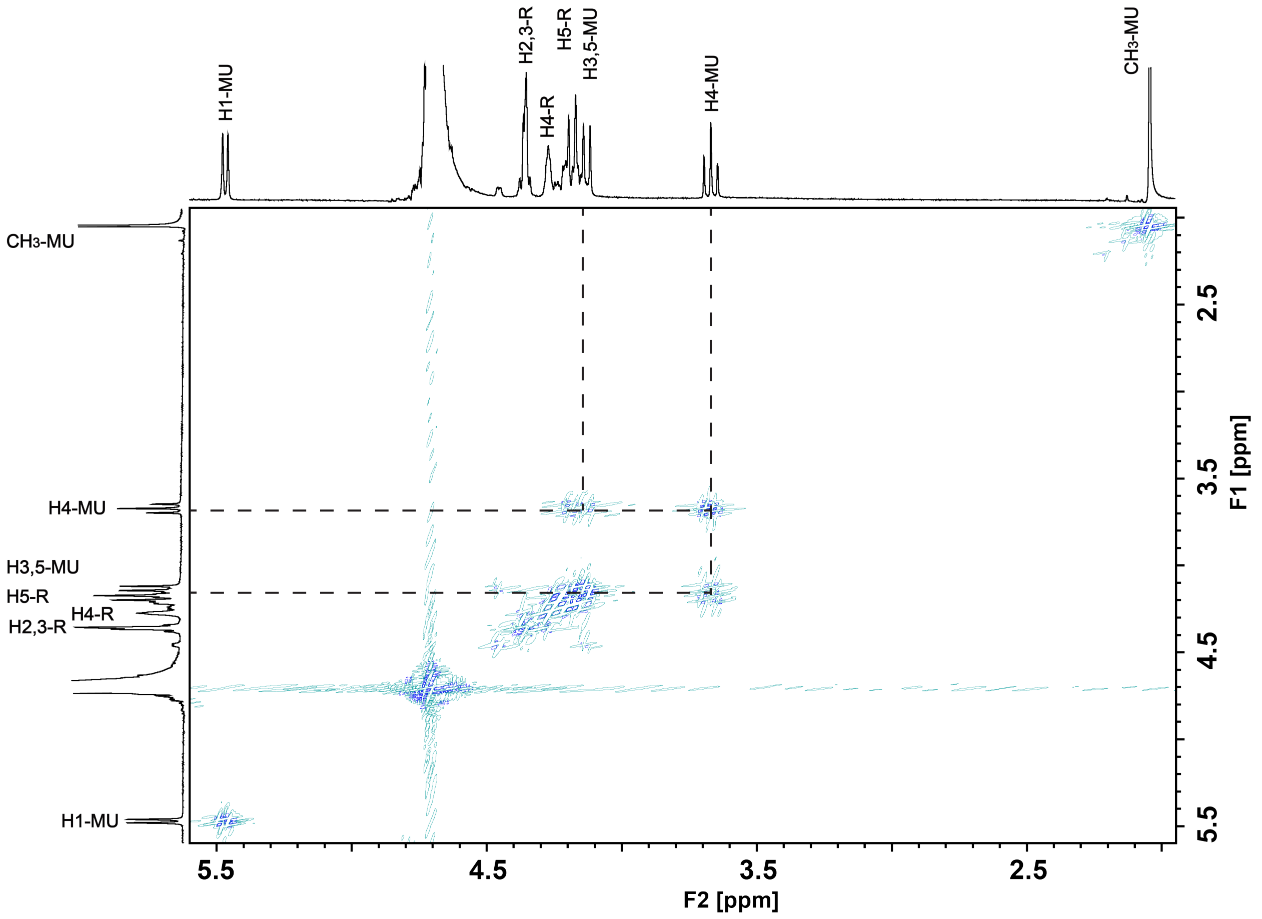

Figure S4. ^1H-^1H COSY NMR spectra of UDP-ManNAcA (**3**) produced in D_2O with the catalytic activities of non-hydrolyzing C2-epimerase and C6-dehydrogenase from serotype HS:11. Resonances for the hydrogens labeled with a "R" correspond to the ribose moiety of UDP, while those labeled with a "MU" correspond to those of the ManNAcA moiety. Additional details are provided in the text.

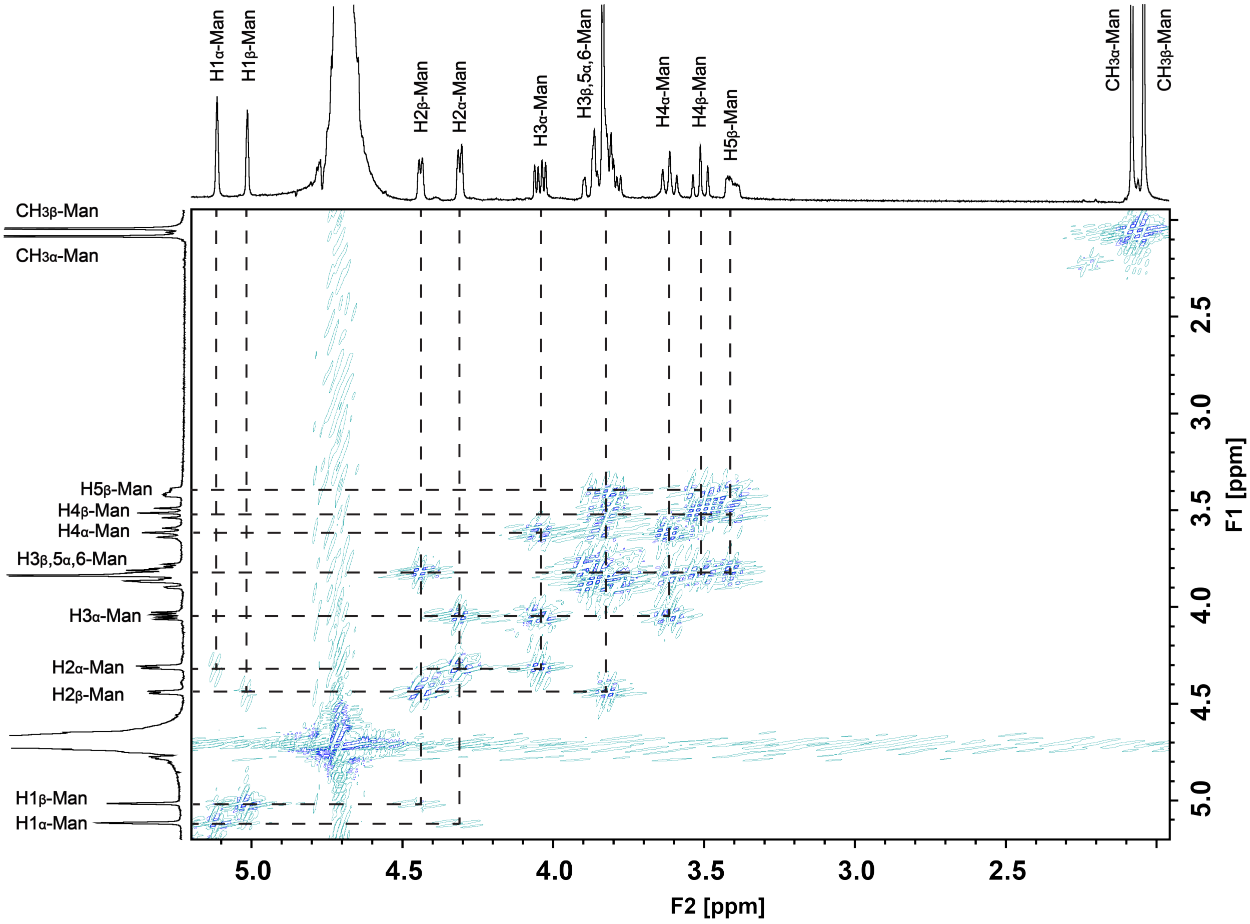

Figure S5. ^1H-^1H COSY NMR spectra of ManNAc (**4**) produced in H_2O with the catalytic activities of hydrolyzing C2-epimerase from serotype HS:6. Resonances for the hydrogens labeled with a "Man" correspond to the *N*-acetyl-D-mannosamine moiety. Additional details are provided in the text.

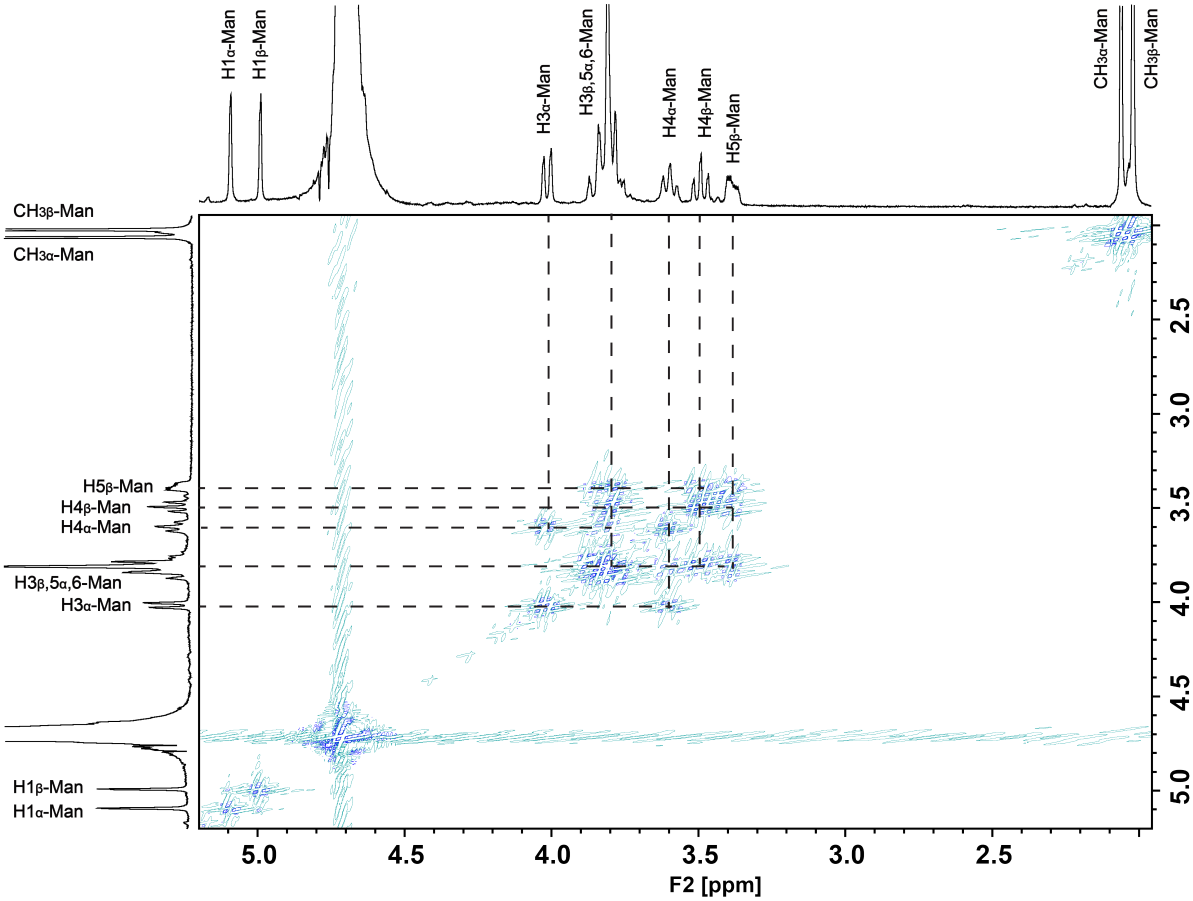

Figure S6. ^1H-^1H COSY NMR spectra of ManNAc (**4**) produced in D_2O with the catalytic activities of hydrolyzing C2-epimerase from serotype HS:6. Resonances for the hydrogens labeled with a "Man" correspond to the *N*-acetyl-D-mannosamine moiety. Additional details are provided in the text.

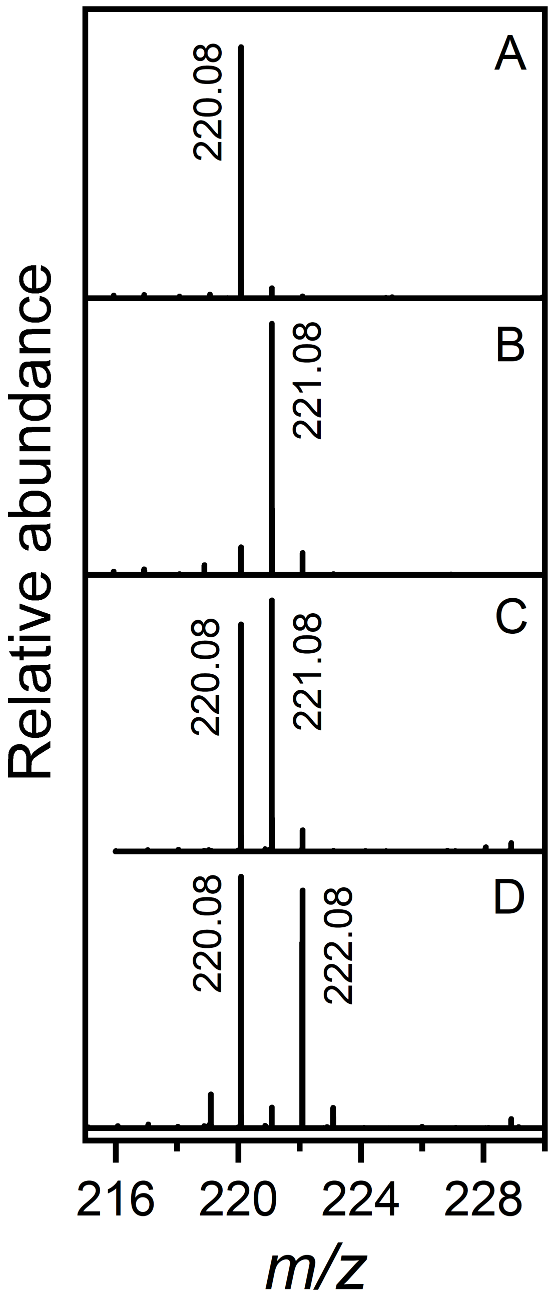

Figure S7. Mass spectrometric analysis of the reaction catalyzed by the hydrolyzing C2-epimerase from serotype HS:6. (A) Reaction conducted in H_2O. (B) Reaction conducted in D_2O. (C) Reaction conducted in 50% D_2O. (D) Reaction conducted in 50% [^{18}O]-H_2O. Additional details are provided in the text.

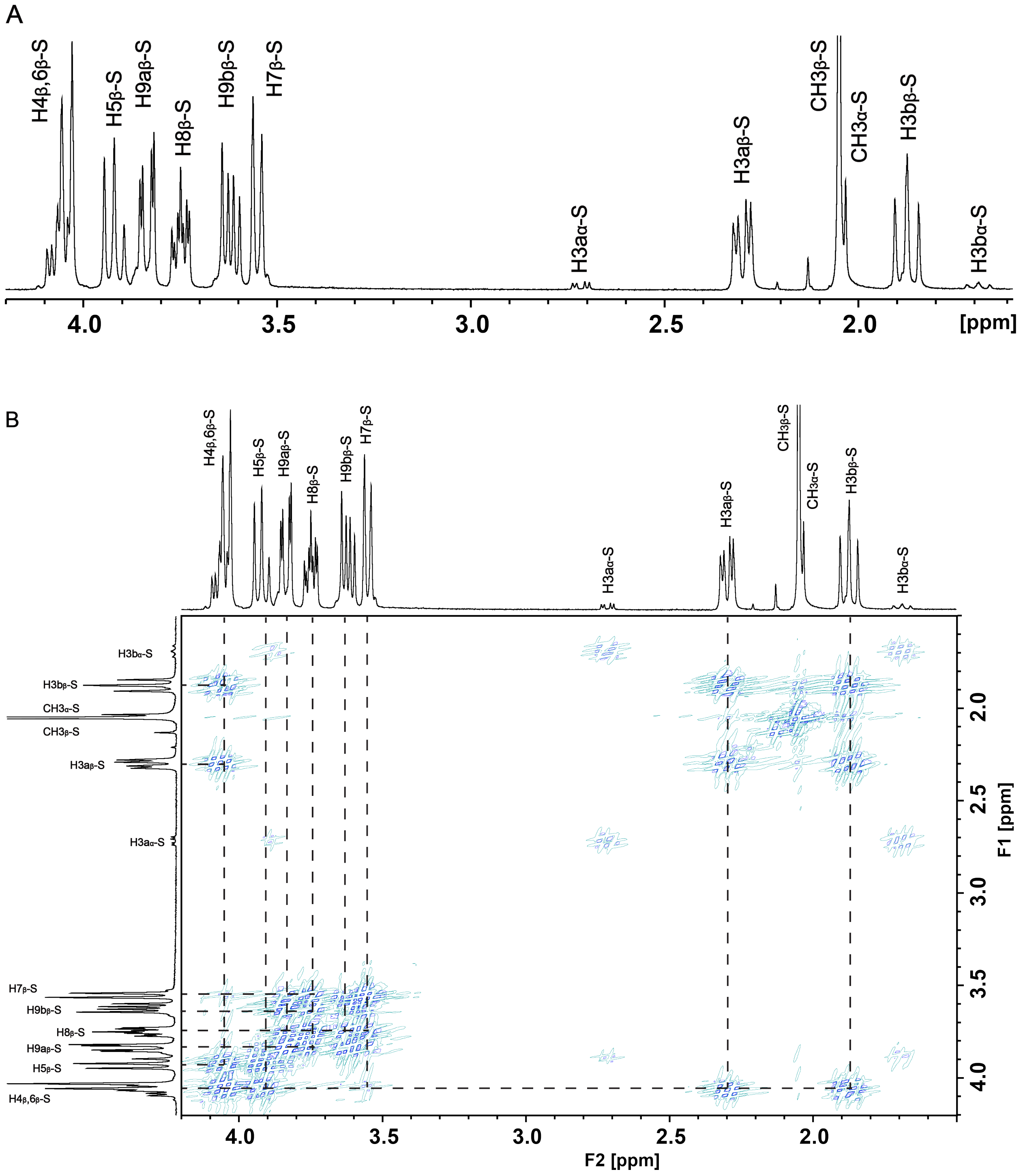

Figure S8. ^{1}H NMR and ^{1}H-^{1}H COSY NMR spectra of *N*-acetylneuraminic acid (**5**) produced from the catalytic activity of the Neu5Ac synthase from serotype HS:6. The reaction was conducted in H_2O. (A) ^{1}H NMR spectra and (B) ^{1}H-^{1}H COSY NMR spectra of *N*-acetylneuraminic acid (**5**). Resonances for the hydrogens labeled with a "S" correspond to the *N*-acetylneuraminic acid moiety. Additional details are provided in the text.

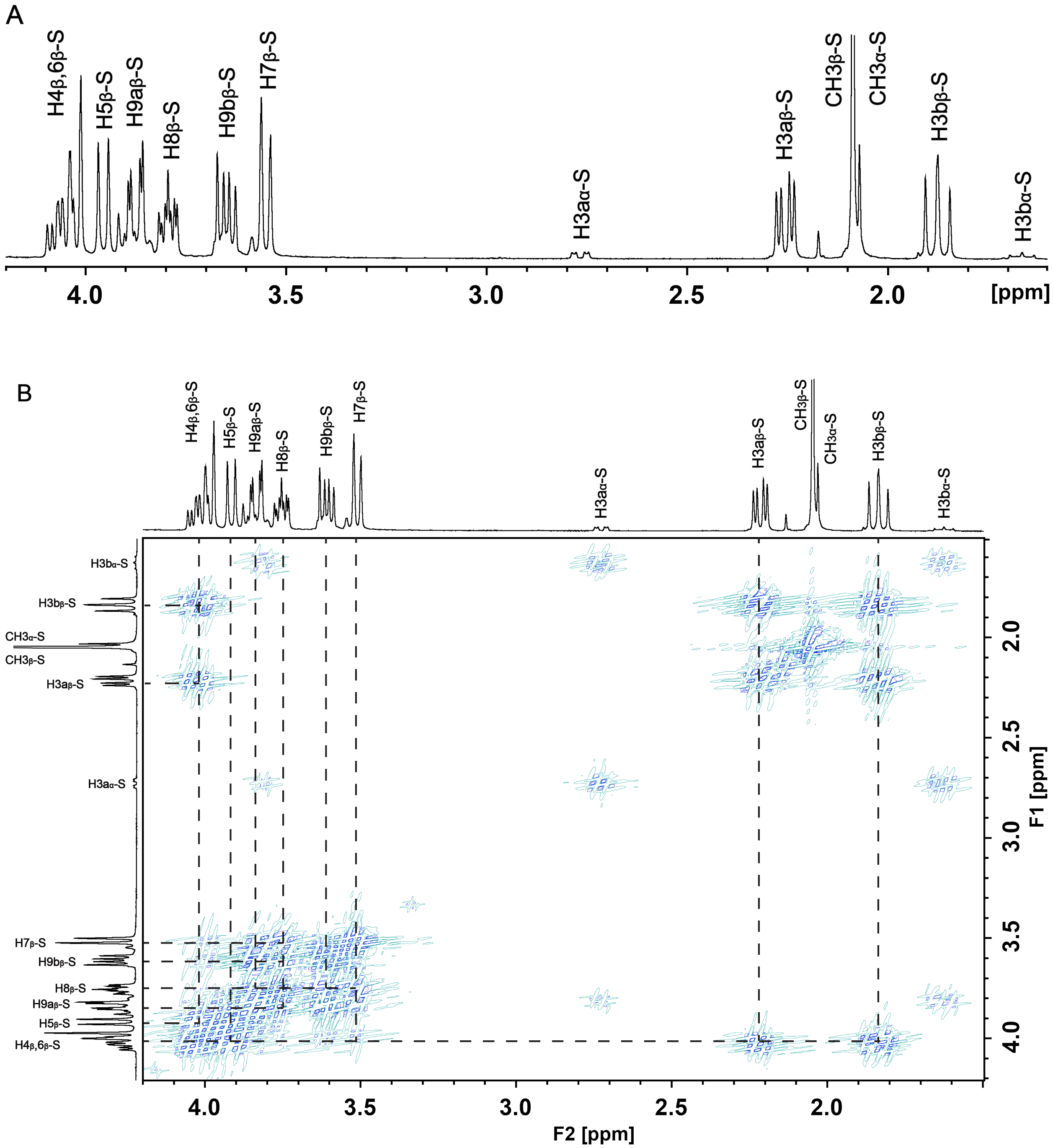

Figure S9. ^{1}H NMR and ^{1}H-^{1}H COSY NMR spectra of *N*-acetylneuraminic acid (**5**) produced from the catalytic activity of the *N*-acetylneuraminate synthase from serotype HS:6. The reaction was conducted in D_2O. (A) ^{1}H NMR spectra and (B) ^{1}H-^{1}H COSY NMR spectra of *N*-acetylneuraminic acid (**5**). Resonances for the hydrogens labeled with a "S" correspond to the *N*-acetylneuraminic acid moiety. Additional details are provided in the text.

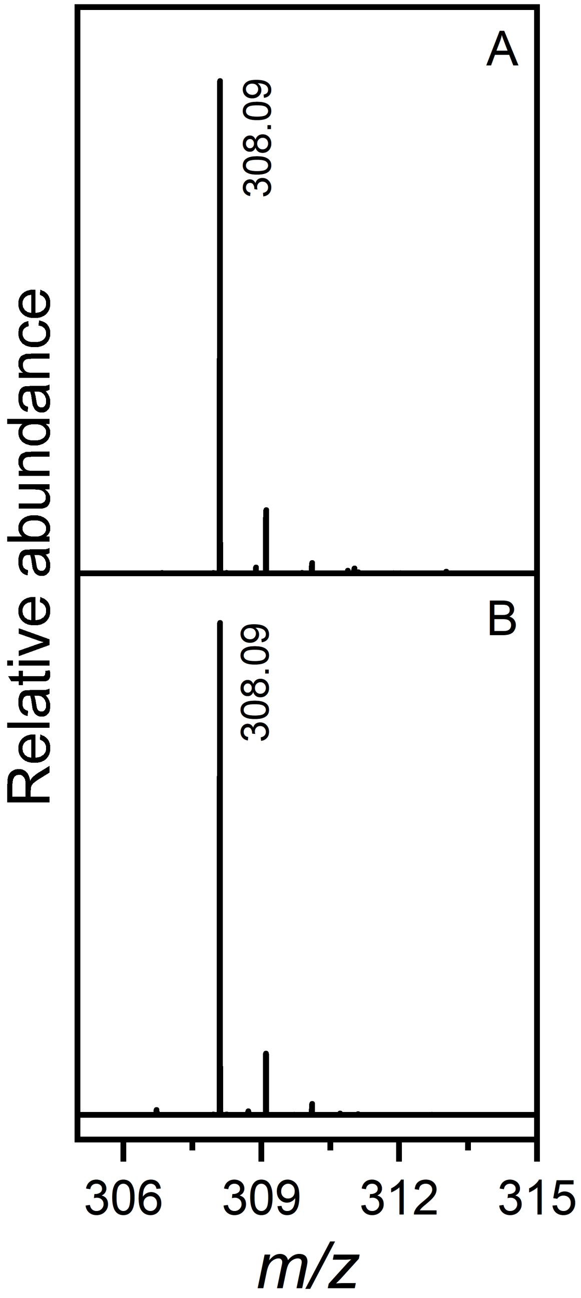

Figure S10. Mass spectrometry analysis of the reaction catalyzed by the *N*-acetylneuraminate synthase from serotype HS:6. (A) Reaction conducted in H_2O. (B) Reaction conducted in D_2O. Additional details are provided in the text.

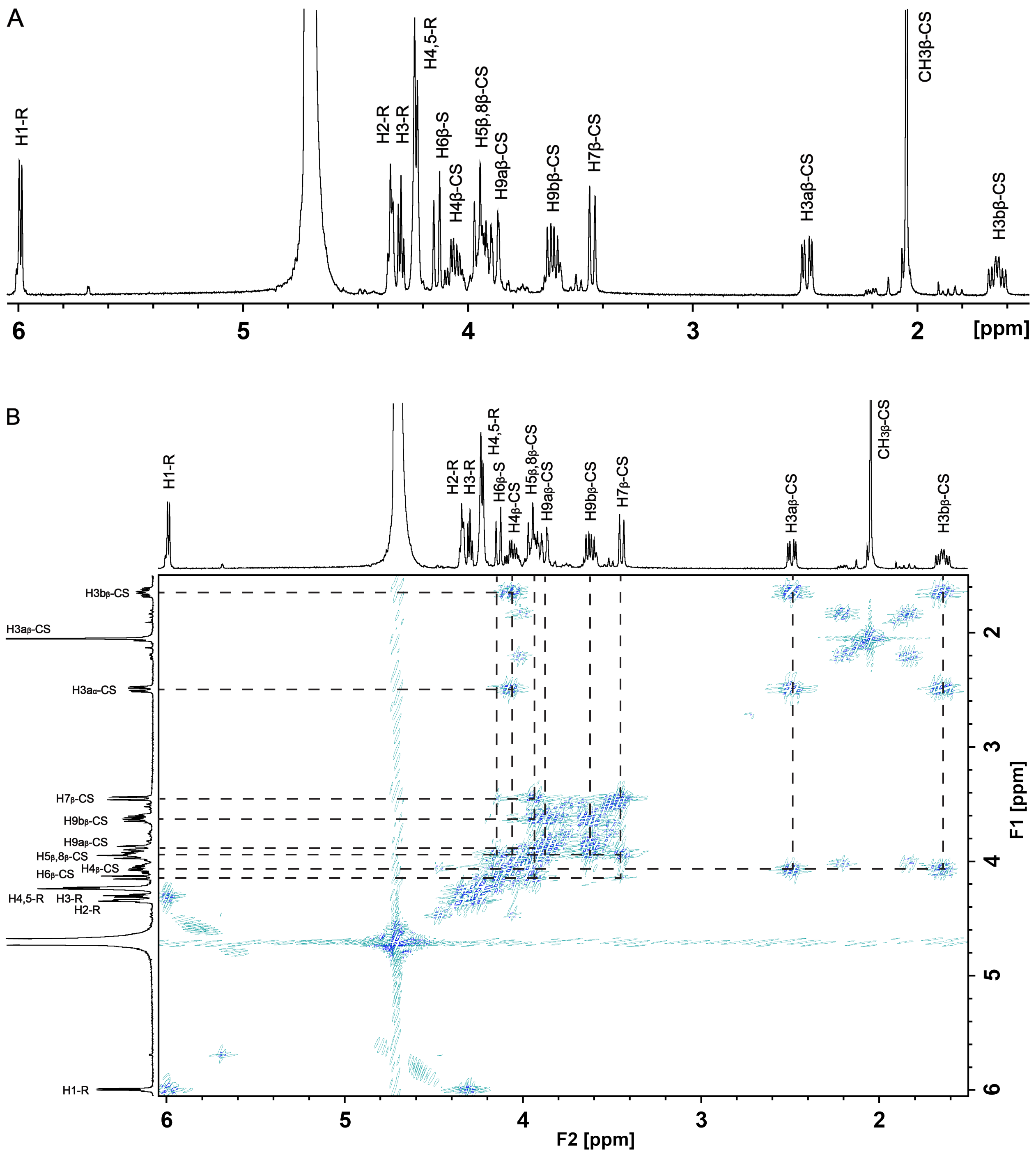

Figure S11. ^1H NMR and ^1H-^1H COSY NMR spectra of CMP-Neu5Ac (**6**) produced from the catalytic activity of the CMP-Neu5Ac synthase from serotype HS:6. The reaction was conducted in H_2O. (A) ^1H NMR spectra and (B) ^1H-^1H COSY NMR spectra of CMP-Neu5Ac (**6**). Resonances for the hydrogens labeled with a "R" correspond to the ribose moiety of CMP, while those labeled with a "CS" correspond to those of the Neu5Ac moiety. Additional details are provided in the text.

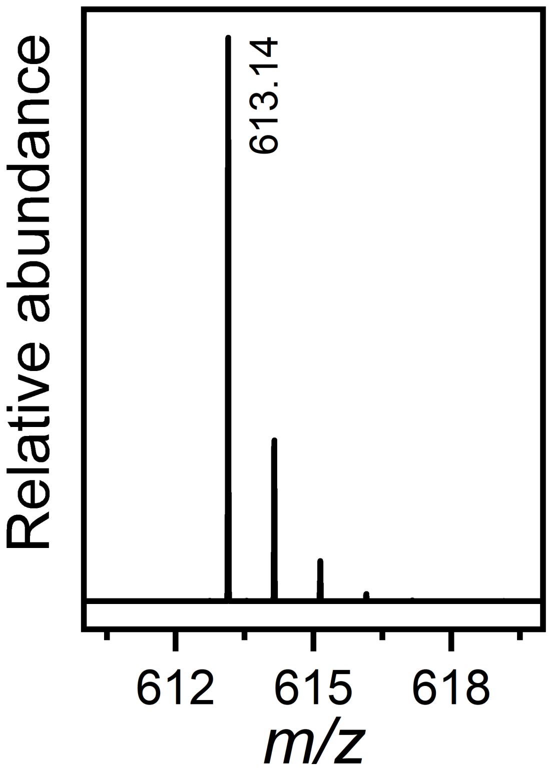

Figure S12. Mass spectrometric analysis of the reaction catalyzed by the CMP-Neu5Ac synthase from serotype HS:6 in H_2O. Additional details are provided in the text.

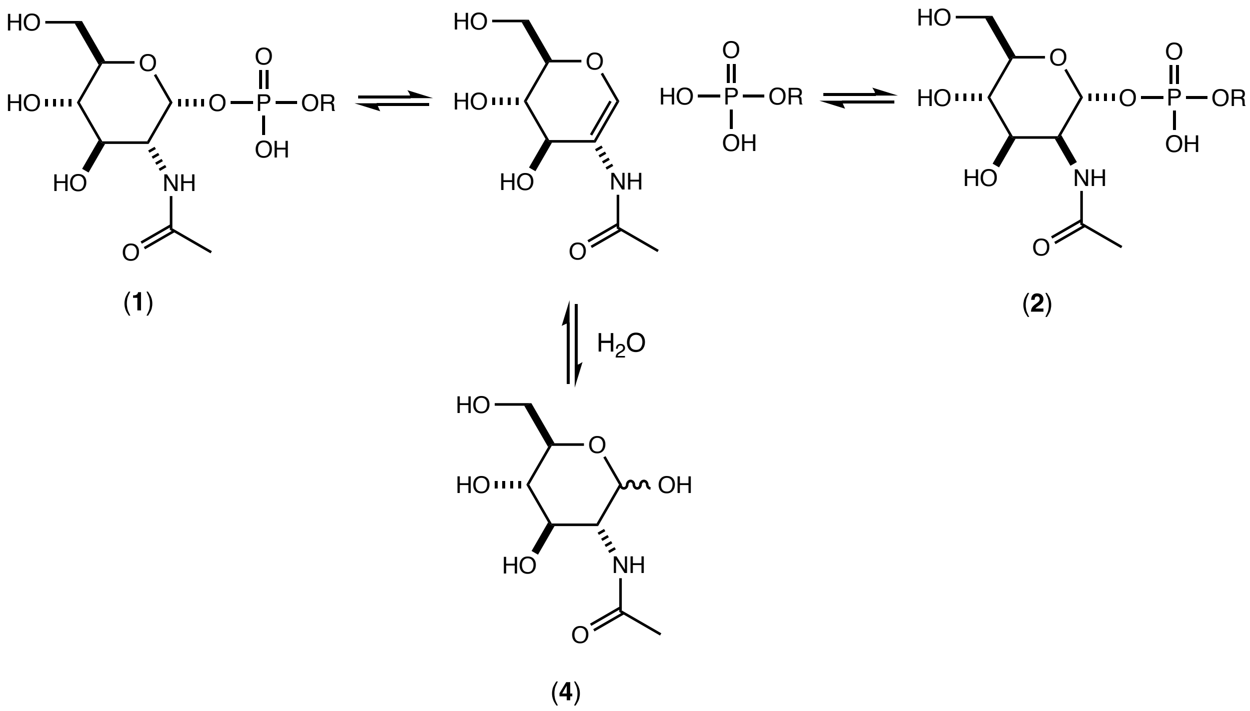

Scheme S1: Proposed reaction mechanisms for the reaction catalyzed by the non-hydrolyzing UDP-GlcNAc C2-epimerase (from **1** to **2**) and the hydrolyzing UDP-GlcNAc C2-epimerase (from **1** to **4**) showing the putative 2-acetamidoglucal intermediate (*1-7*).

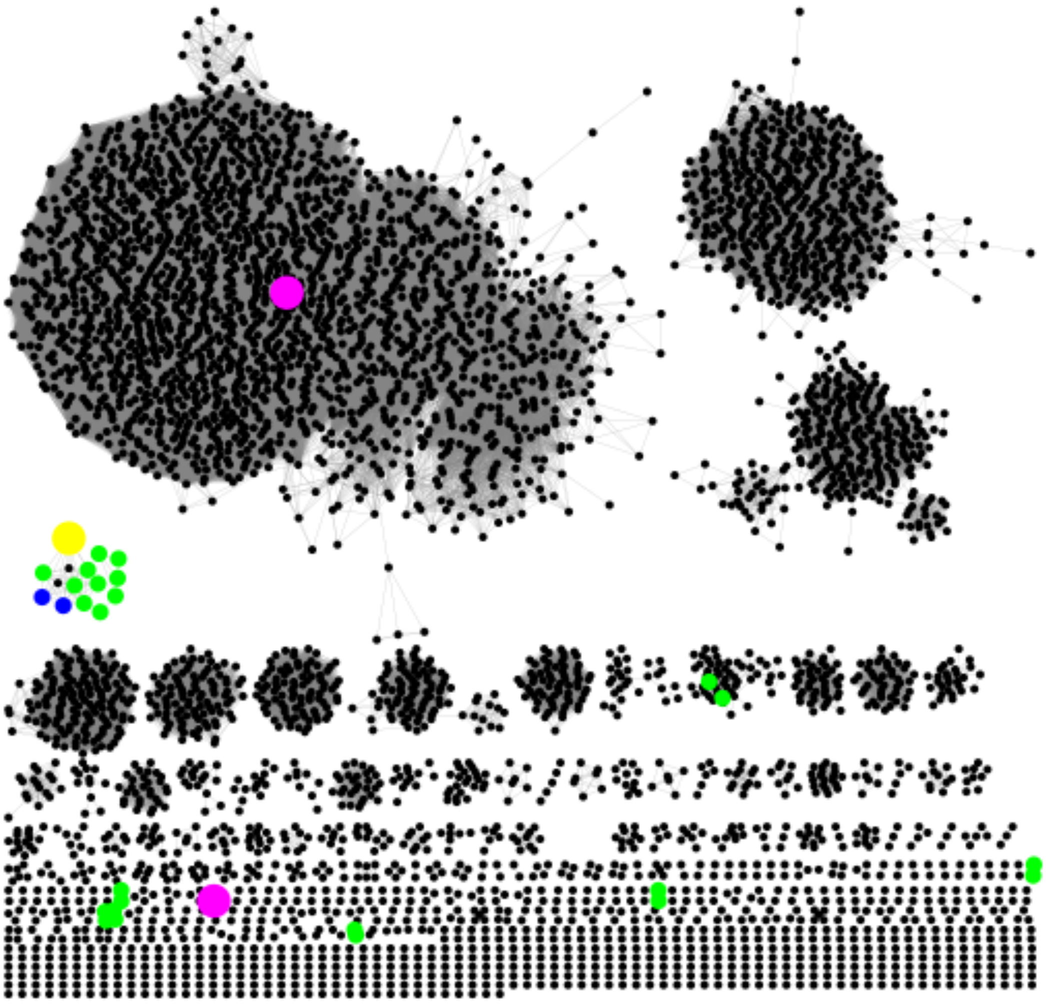

Figure S13. SSN for the non-hydrolyzing C2-epimerase from *C. jejuni* serotype HS:11. The closest 5000 sequences to the non-hydrolyzing C2-epimerase from *C. jejuni* serotype HS:11 at a sequence identity cutoff of 59%. The sequences for the functionally characterized non-hydrolyzing C2-epimerases from *E. coli* K12 and *N. meningitidis* DSM15465 are shown in pink. The green and blue circles represent the putative non-hydrolyzing C2-epimerases from various *Campylobacter* species and *C. jejuni*, respectively. The yellow circle represents the non-hydrolyzing C2-epimerase from serotypes HS:11.

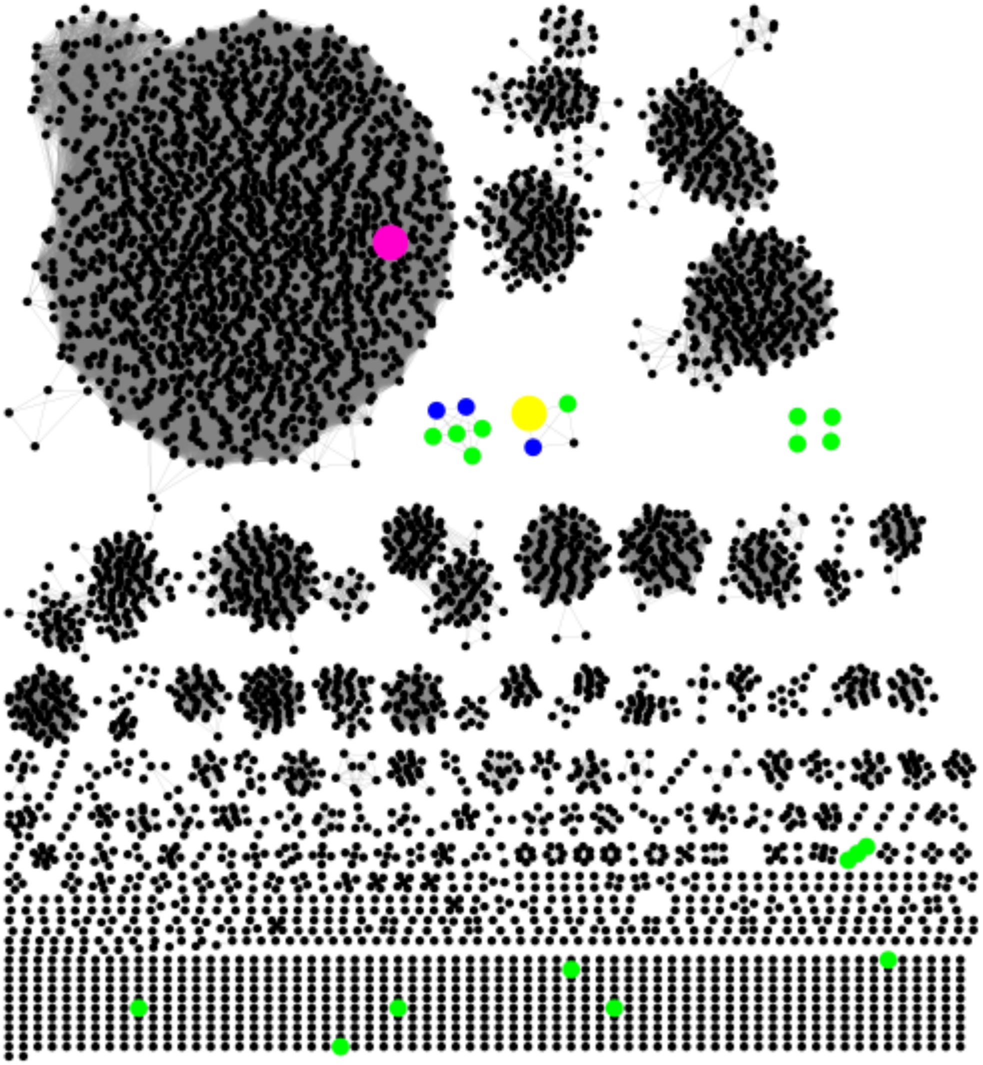

Figure S14. SSN for the UDP-ManNAc C6-dehydrogenase from *C. jejuni* serotype HS:11. The closest 5000 sequences to the C6-dehydrogenase from *C. jejuni* serotype HS:11 at a sequence identity cutoff of 73%. The sequence for the C6-dehydrogenase from *E. coli* K12 is shown in pink. The green and blue circles represent the putative C6-dehydrogenases from various *Campylobacter* species and *C. jejuni*, respectively. The yellow circle represents C6-dehydrogenase from serotypes HS:11.

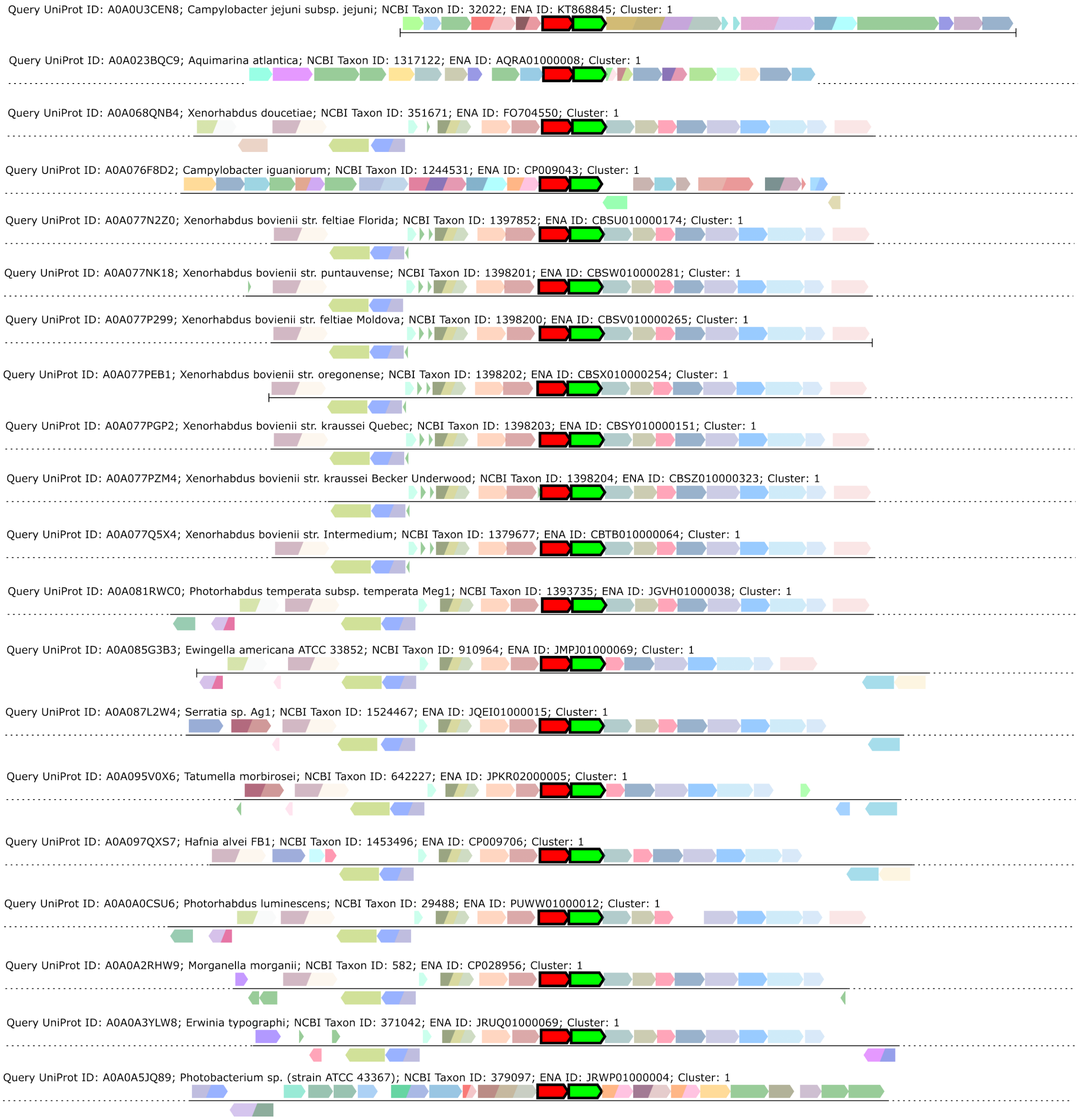

Figure S15. A portion of the genome neighborhood network (GNN) for the UDP-ManNAcA (**3**) biosynthetic pathway. Network is aligned based on the non-hydrolyzing C2-epimerases (Pfam identifier 02350) from *C. jejuni* serotype HS:11 homologs (red color) and the UDP-ManNAc C6-dehydrogenases from *C. jejuni* serotype HS:11 homologs (Pfam identifier 00984) (colored green).

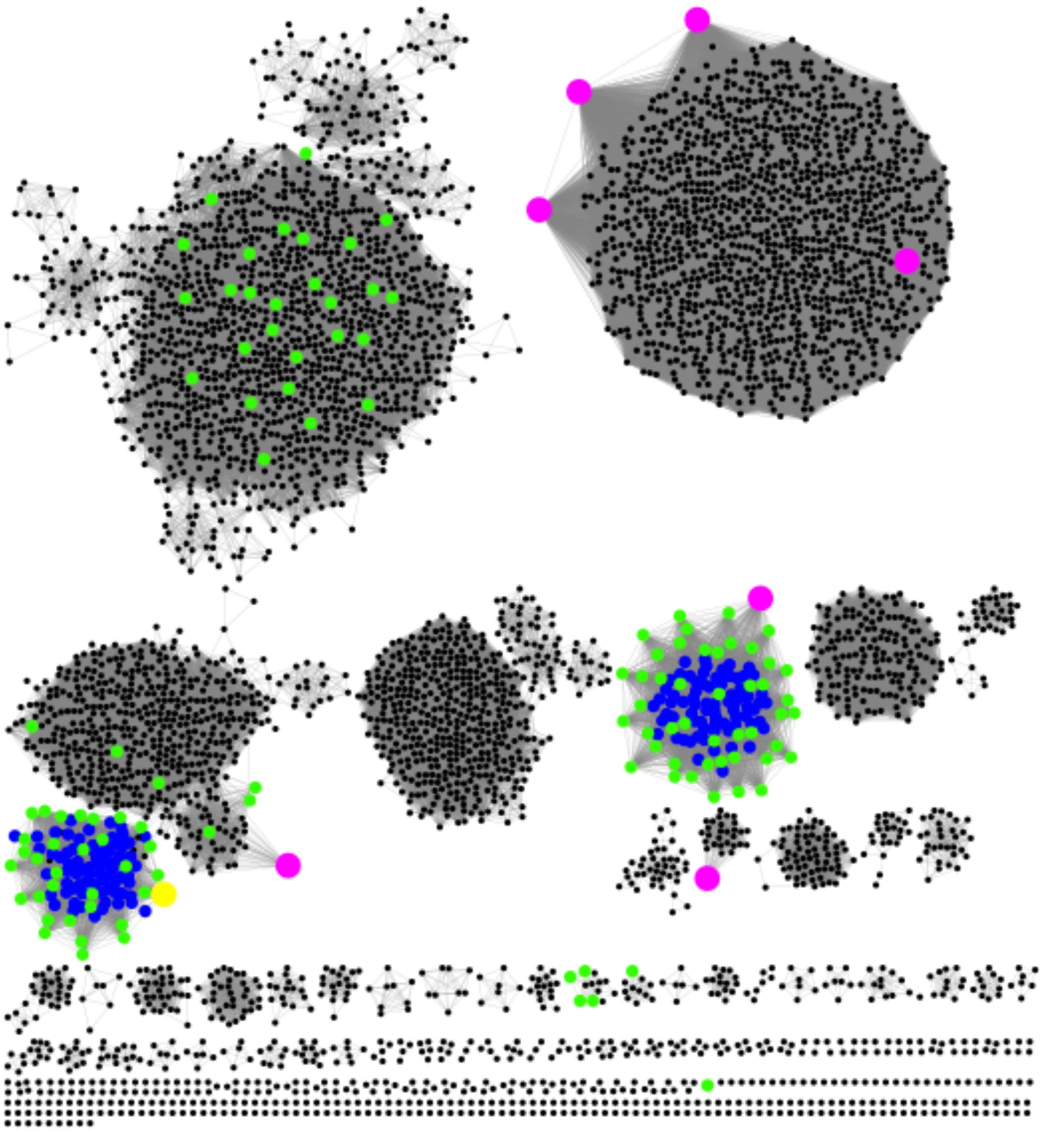

Figure S16. SSN for the hydrolyzing UDP-GlcNAc C2-epimerase from *C. jejuni* serotype HS:6. The closest 5000 sequences to the hydrolyzing C2-epimerase from *C. jejuni* serotype HS:6 at a sequence identity cutoff of 50%. The sequences for the functionally characterized hydrolyzing C2-epimerases are shown in pink. The green and blue circles represent the putative hydrolyzing C2-epimerases from various *Campylobacter* species and *C. jejuni*, respectively. The yellow circle represents the hydrolyzing C2-epimerase from serotypes HS:6.

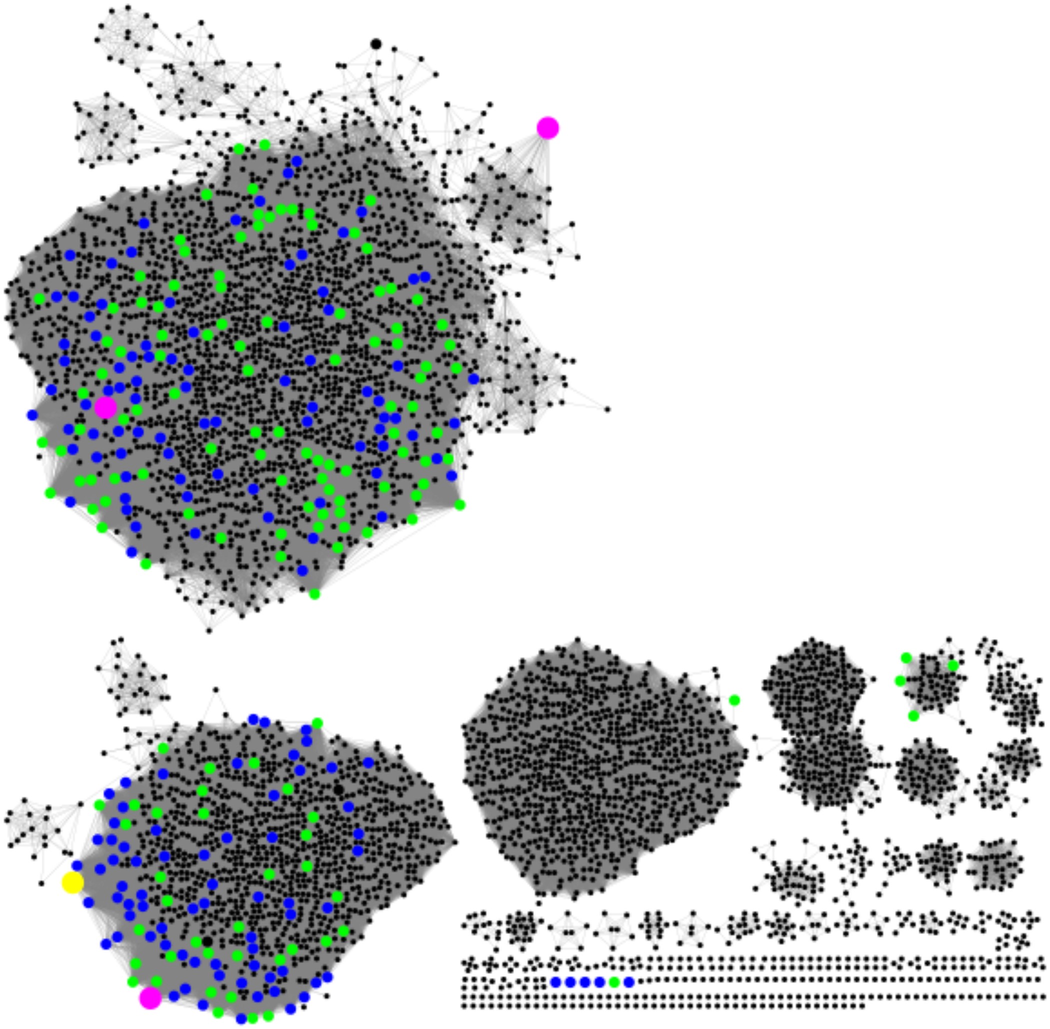

Figure S17. SSN for the Neu5Ac synthase from *C. jejuni* serotype HS:6. The closest 5000 sequences to the Neu5Ac synthase from *C. jejuni* serotype HS:6 at a sequence identity cutoff of 50%. The sequences for the functionally characterized Neu5Ac synthases are shown in pink. The green and blue circles represent the putative *N*-acetylneuraminate synthases from various *Campylobacter* species and *C. jejuni*, respectively. The yellow circle represents the Neu5Ac synthase from serotypes HS:6.

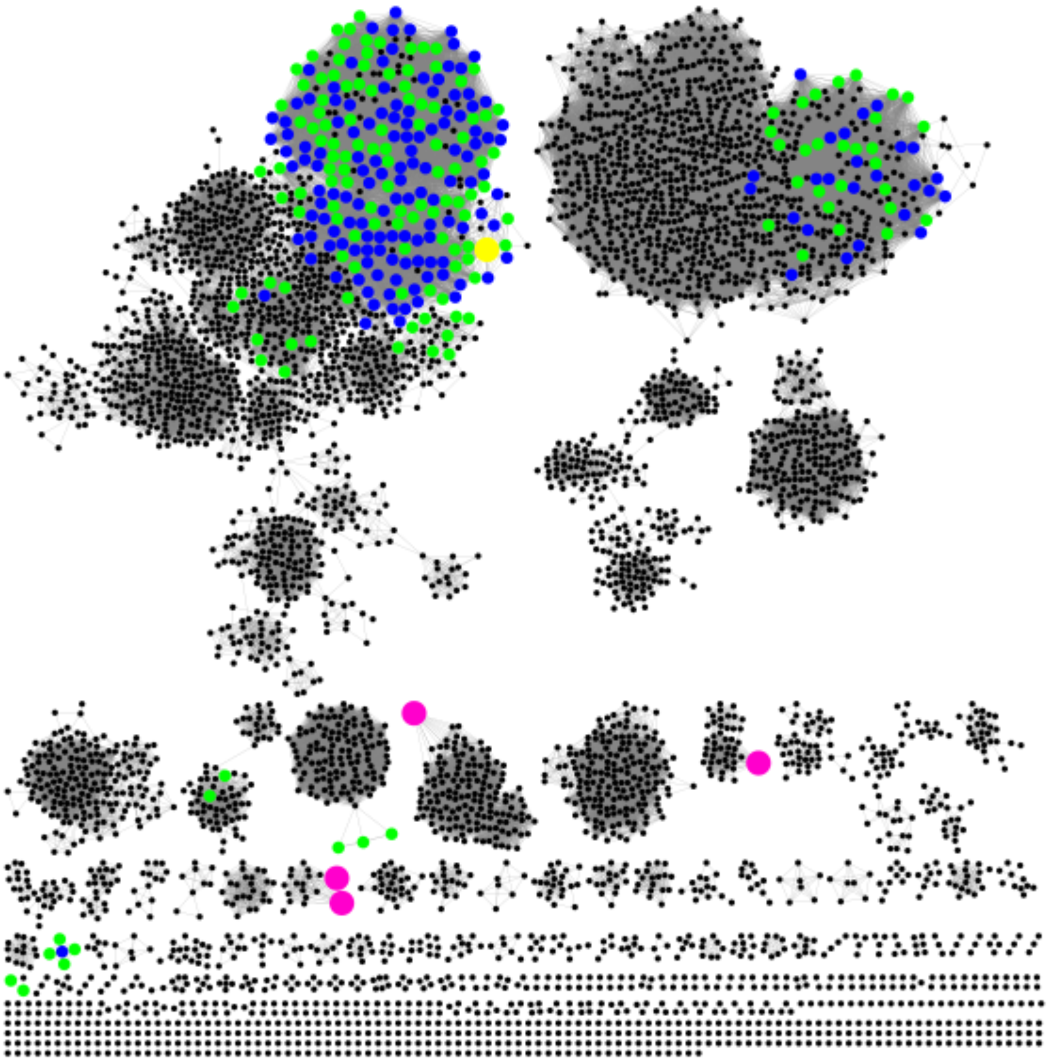

Figure S18. SSN for the CMP-Neu5Ac synthase from *C. jejuni* serotype HS:6. The closest 5000 sequences to the CMP-Neu5Ac synthases from *C. jejuni* serotype HS:6 at a sequence identity cutoff of 55%. The sequences for the functionally characterized CMP-Neu5Ac synthases are shown in pink. The green and blue circles represent the putative CMP-Neu5Ac synthases from various *Campylobacter* species and *C. jejuni*, respectively. The yellow circle represents the CMP-Neu5Ac synthase from serotypes HS:6.

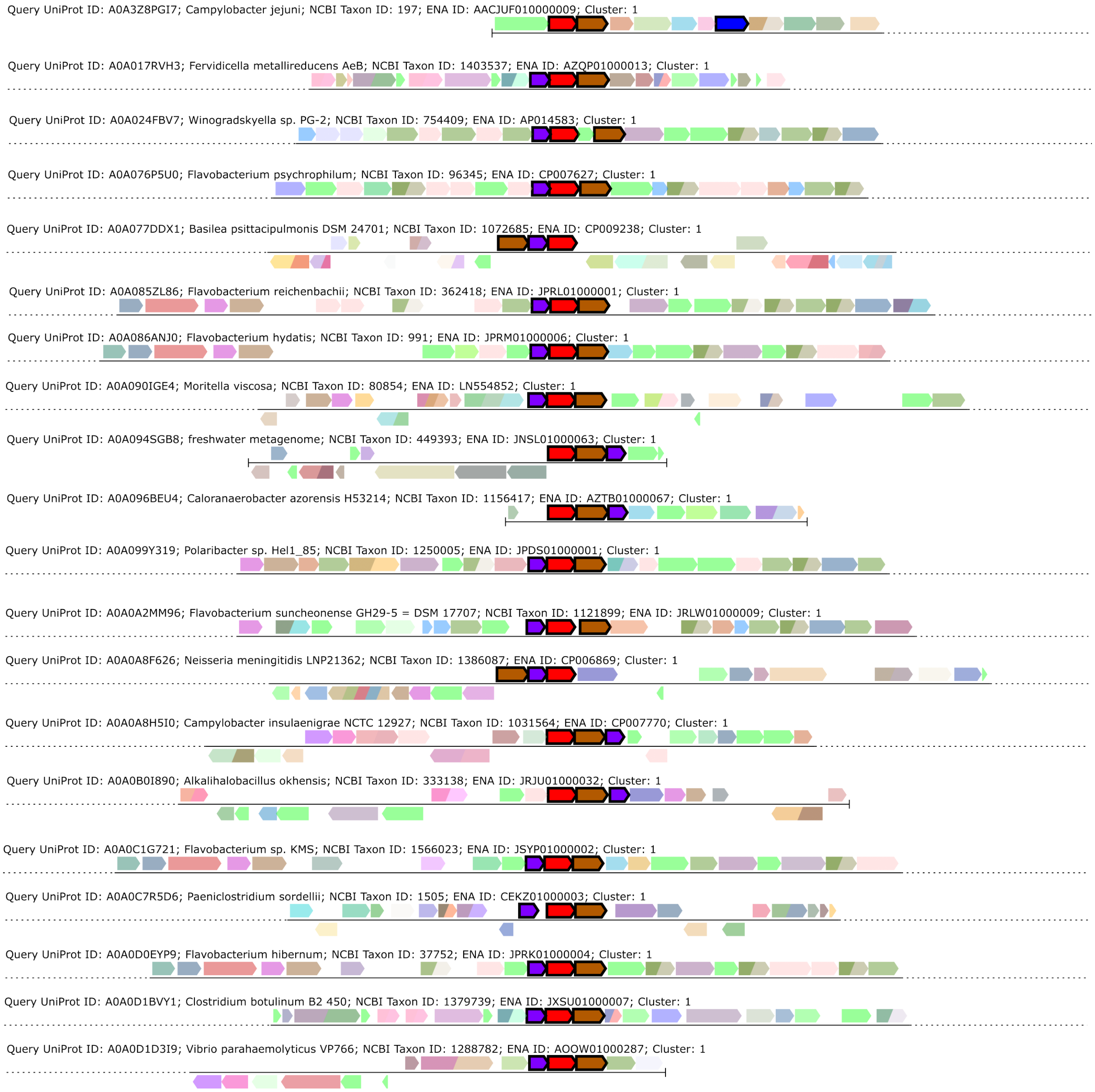

Figure S19. A portion of the genome neighborhood network (GNN) for the CMP-Neu5Ac (**6**) biosynthetic pathway. Network is aligned based on hydrolyzing C2-epimerase (Pfam identifier 02350; brown color), the *N*-acetylneuraminate synthase (Pfam identifier 03102; red color) and the CMP-Neu5Ac synthase (Pfam identifier 02348; blue color) from *C. jejuni* serotype HS:6.

REFERENCES

1. Sala, R. F.; Morgan, P. M.; and Tanner, M. E. Enzymatic Formation and Release of a Stable Glycal Intermediate: The Mechanism of the Reaction Catalyzed by UDP-*N*-Acetylglucosamine 2-Epimerase. *J. Am. Chem. Soc.* 1996, *118*, 3033-3034.

2. Morgan, P. M.; Sala, R. F.; and Tanner, M. E. Eliminations in the Reactions Catalyzed by UDP-*N*-Acetylglucosamine 2-Epimerase. *J. Am. Chem. Soc.* 1997, *119*, 10269-10277.

3. Samuel, J.; and Tanner, M. E. Active site mutants of the "non-hydrolyzing" UDP-*N*-acetylglucosamine 2-epimerase from *Escherichia coli*. *Biochim. Biophys. Acta* 2004, *1700*, 85-91.

4. Vann, W. F.; Daines, D. A.; Murkin, A. S.; Tanner, M. E.; Chaffin, D. O.; Rubens, C. E.; Vionnet, J.; and Silver, R. P. The NeuC protein of *Escherichia coli* K1 is a uridine diphosphate N-acetylglucosamine 2-epimerase. *J. Bacteriol.* 2004, *186*, 704-712.

5. Murkin, A. S.; Chou, W. K.; Wakarchuk, W. W.; and Tanner, M. E. Identification and mechanism of a bacterial hydrolyzing UDP-N-acetylglucosamine 2-epimerase *Biochemistry* 2004, *43*, 14290-14298.

6. Hinderlich, S.; Stasche, R.; Zeitler, R.; and Reutter, W. A Bifunctional Enzyme Catalyzes the First Two Steps in *N*-Acetylneuraminic Acid Biosynthesis of Rat Liver. *J. Biol. Chem.* 1997, *272*, 24313-24318.

7. Chou, W. K.; Hinderlich, S.; Reutter, W.; and Tanner, M. E. Sialic acid biosynthesis: stereochemistry and mechanism of the reaction catalyzed by the mammalian UDP-N-acetylglucosamine 2-epimerase. *J. Am. Chem. Soc.* 2003, *125*, 2455-2461
